# Tertiary lymphoid structures in diseases: immune mechanisms and therapeutic advances

**DOI:** 10.1038/s41392-024-01947-5

**Published:** 2024-08-28

**Authors:** Lianyu Zhao, Song Jin, Shengyao Wang, Zhe Zhang, Xuan Wang, Zhanwei Chen, Xiaohui Wang, Shengyun Huang, Dongsheng Zhang, Haiwei Wu

**Affiliations:** 1 Department of Oral and Maxillofacial Surgery, Shandong Provincial Hospital Affiliated to Shandong First Medical University, Jinan, Shandong China; 2 School of Stomatology, Shandong First Medical University, Jinan, China; 3 Department of Oral and Maxillofacial Surgery, School and Hospital of Stomatology, Cheeloo College of Medicine, Shandong University & Shandong Key Laboratory of Oral Tissue Regeneration & Shandong Engineering Laboratory for Dental Materials and Oral Tissue Regeneration & Shandong Provincial Clinical Research Center for Oral Diseases, Jinan, Shandong China

**Keywords:** Immunotherapy, Applied immunology

## Abstract

Tertiary lymphoid structures (TLSs) are defined as lymphoid aggregates formed in non-hematopoietic organs under pathological conditions. Similar to secondary lymphoid organs (SLOs), the formation of TLSs relies on the interaction between lymphoid tissue inducer (LTi) cells and lymphoid tissue organizer (LTo) cells, involving multiple cytokines. Heterogeneity is a distinguishing feature of TLSs, which may lead to differences in their functions. Growing evidence suggests that TLSs are associated with various diseases, such as cancers, autoimmune diseases, transplant rejection, chronic inflammation, infection, and even ageing. However, the detailed mechanisms behind these clinical associations are not yet fully understood. The mechanisms by which TLS maturation and localization affect immune function are also unclear. Therefore, it is necessary to enhance the understanding of TLS development and function at the cellular and molecular level, which may allow us to utilize them to improve the immune microenvironment. In this review, we delve into the composition, formation mechanism, associations with diseases, and potential therapeutic applications of TLSs. Furthermore, we discuss the therapeutic implications of TLSs, such as their role as markers of therapeutic response and prognosis. Finally, we summarize various methods for detecting and targeting TLSs. Overall, we provide a comprehensive understanding of TLSs and aim to develop more effective therapeutic strategies.

## Introduction

Tertiary lymphoid structures (TLSs) are organized, non-encapsulated aggregates of lymphoid cells that form in non-lymphoid tissues under pathological conditions after birth.^1,2^ TLSs mainly include germinal centers (GCs), surrounding T-cell areas, and distributed PANd^+^ high endothelial venules (HEVs).^3–5^ TLSs typically form in response to chronic inflammation, such as infections, autoimmune diseases, tissue transplants, and cancers.^6–12^ Recent studies have found a positive correlation between the presence of TLSs and the efficacy of immune checkpoint blockade (ICB) therapy. However, the underlying mechanism remains unclear, and only a minority of patients benefit from this therapy.^6,13,14^ Moreover, the prognostic value of TLSs has been widely discussed. In conditions that require an enhanced immune response, such as cancer and infectious diseases, the presence and maturation of TLSs generally indicate more favorable outcomes.^6,15–19^ Conversely, in autoimmune diseases and age-related chronic inflammatory conditions, which involve the immune system attacking self-tissues, the emergence and maturation of TLSs are often associated with poorer prognoses.^20–22^ Therefore, TLSs can be seen as a double-edged sword. To fully harness the potential of TLSs, a comprehensive understanding of TLSs is urgently needed.

**Fig. 1:**
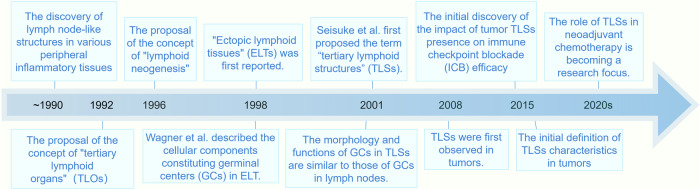
Milestone events of the discovery and development of TLSs. Key milestones in tertiary lymphoid structures are indicated. In this figure, we summarize the concept of Tertiary Lymphoid Structures (TLSs), their discovery process, and the progress in research. TLSs are aggregates of lymphoid cells that form in non-lymphoid tissues under pathological conditions. The concept was formally introduced in 1992, and the term “TLSs” was coined in 2001. Over the past decade, TLSs have gained significant attention and have been widely studied. By Figdraw

In 1964, Ziff noticed similarities between inflamed rheumatoid synovial tissue and lymph nodes, where the primary immune response occurs.^23,24^ In the early 1970s, Söderström et al. found that the structure of thyroid tissue in patients with Hashimoto’s thyroiditis resembled that of lymph nodes.^24,25^ In 1992, Louis Picker and Eugene Butcher formally introduced the concept of “tertiary lymphoid organs” (TLOs) or “tertiary lymphoid tissues” (TLTs). At that time, it was believed that TLOs could occur in all tissues of the body, except primary lymphoid organs (bone marrow and thymus) and secondary lymphoid organs (lymph nodes, spleen, and gut-associated lymphoid tissue). TLOs were considered sites where memory lymphocytes and effector precursor cells could be re-stimulated by antigens, or where B cells and T cells could carry out terminal responses. However, they believed that TLOs are an unorganized structures composed of a small number of lymphocytes. If long-term stimulation leads to the formation of lymph node-like structures in TLOs, they have transformed into secondary lymphoid structures.^9,26^ In 1996, Kratz et al. demonstrated that lymphotoxin could induce the formation of lymphoid tissues in chronic inflammatory conditions. This is similar to the induction signals for lymphoid organogenesis during embryonic development. Thus, they termed the de novo formation process of organized lymphoid tissue “lymphoid neogenesis” in chronic inflammation.^27,28^ In 1998, Wagner et al. proposed the concept of “ectopic lymphoid tissues” (ELTs), because patients with rheumatoid synovitis had follicle-like structures in their synovial tissues that resembled secondary lymphoid follicles. They also clarified the cellular components required for GC formation within it.^29^ In 2001, Seisuke et al. discovered GCs in rheumatoid synovitis with the same morphology and function as lymph node GCs. Thus, they proposed the term “tertiary lymphoid structures” (TLSs).^30^ In 2015, Jin et al. described the characteristics of TLSs in breast cancer.^31^ Some studies also refer to TLSs as ectopic lymphoid organs or ectopic lymphoid structures.^32,33^ In the first decade of the 21st century, research on TLSs mainly focused on chronic inflammation, autoimmune diseases, and immune rejection after organ transplantation. In 2008, TLSs were reported to be associated with a better prognosis in non-small cell lung cancer, marking the first discovery of TLSs in tumors.^34^ ICB has been a focal point in tumor treatment, and in 2015, Giraldo et al. first linked the presence of TLSs with the efficacy of ICB therapy.^35^ Subsequent research further confirmed that the presence of TLSs can predict the positive effects of ICB treatment.^36–38^ Since 2020, this topic has received wider attention and has become a hot topic in the field of TLS research.^6,12,39^ However, our understanding of TLSs is still very limited. (Fig. 1).

In this review, we focused on the composition and cascade regulatory mechanisms of TLSs. Furthermore, we summarize the influence of TLSs on common diseases and ageing, especially primary and metastatic tumors. Based on these knowledge, we discuss the potential application of TLSs as predictors of prognosis and targets for immunetherapy. Finally, we explore the future research directions and challenges in the study of TLSs, aiming to provide a comprehensive and in-depth understanding of their scientific significance and clinical implications.

## The cellular composition of TLS

### Stromal cell

**Fig. 2:**
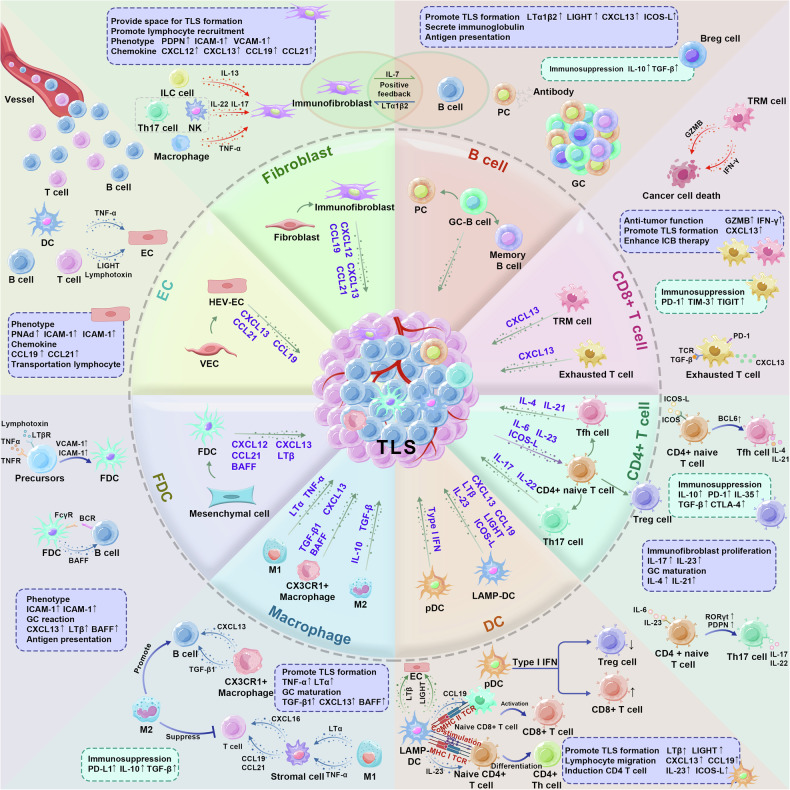
The composition of TLS. TLSs mainly include T cells, B cells, fibroblasts, DCs, macrophages, FDCs, and HEV-EC. Stromal cells undergo phenotypic polarization in the TME, and they produce cytokines and chemokines, which induce aggregation and differentiation of immune cells and promote the formation of TLSs. TLSs have dual effects of pro-tumor and anti-tumor roles, which depend on the internal immune cell subtype

Stromal cells, including fibroblasts, endothelial cells, and follicular dendritic cells (FDCs), are vital for the formation of TLSs. They create a conducive microenvironment for TLS formation, promoting the accumulation and functional activation of immune cells. (Fig. 2).

#### Fibroblast

Fibroblasts provide a space for TLS formation. They promote immune cell survival and aggregation in TLSs through inflammatory cytokines and chemokines, such as B cell activating factor (BAFF), transforming growth factor-β (TGF-β), IL-7, C-X-C motif ligand (CXCL) 13, CXCL12, C-C motif chemokine ligand (CCL) 19, and CCL21.^9,40^ In primary Sjӧgren’s syndrome, podoplanin^+^fibroblast activation protein 1^+^ (PDPN^+^FAP^+^) fibroblasts express platelet-derived growth factor receptor (PDGFR) α, PDGFRβ, intercellular adhesion molecule 1 (ICAM-1), vascular cell adhesion molecule 1 (VCAM-1), mucosal vascular address-seeking protein cell adhesion molecule 1 (MadCAM), and receptor activator for NF-κB ligand (RANK-L) in TLSs.^41^ Chen et al. also identified two types of fibroblasts in bladder urothelial carcinoma: PDGFRα^+^ fibroblasts and regulator of G protein signaling 5^+^ (RGS5^+^) fibroblasts. The former express multiple cytokines and chemokines, including CXCL12, IL-6, CXCL14, CXCL1, and CXCL2, increasing immune cell infiltration.^42^ Additionally, Thy1^+^FAP^+^ fibroblasts significantly increased in melanoma, and their density in TLSs was higher than tumor parenchyma.^43^ Notably, fibroblasts from different sites or diseases appear to exhibit different functions in lymphocyte recruitment,^10^ probably reflecting differences in the activation signals or origination. Overall, the cross-talk between fibroblasts and immune cells leads to the production of cytokines and chemokines, creating favorable conditions for TLS formation.

#### Follicular dendritic cell

The presence of FDCs in GC is a marker of mature TLSs.^44^ They originate from local mesenchymal cells rather than hematopoietic cells, such as perivascular mural cell precursors and marginal reticular precursors.^45^ Additionally, FDCs seem to constitute a specialized subset of myofibroblasts, which derive from bone marrow stromal cell progenitor, expressing αSMA.^46^ FDCs interact with lymphoid tissue inducer (LTi) cells (especially B cells) and other stromal cells, promoting their early stages of development.^47^ The lymphotoxin β receptor (LTβR) initiates and maintains FDCs, and then tumor necrosis factor receptor (TNFR) 1 signaling further matures them. FDCs act as antigen-presenting cells (APCs) and are an essential component of the GC response.^48^ They express VCAM-1 and ICAM-1, complement and FcR (a receptor mediating antigen-antibody responses), delivering natural antigens to B cells by attaching antigens to the synapses (typically antigens captured by complement or antibodies).^49,50^ Moreover, FDCs promote GC formation and TLS mature by secreting cytokines and chemokines, such as CXCL12, CXCL13, IL-6, IL-7, BAFF, and TNF-α.^45^ In TLSs within giant cell arteritis, FDCs coexist with T cells and B cells, accompanied by high expression of CXCL13, CXCR5, CCL21, CCR7, LTβ, and BAFF.^51^ And deleting FDCs reduced B cells and impaired GC formation.^52^

### B lymphocyte subsets

Different B cell subsets are associated with TLSs, including plasma cells (PCs), regulatory B (Breg) cells and memory B cells.^53^ They have been reported in many diseases, such as tumors,^44,54^ autoimmunity diseases,^55,56^ and organ transplantation. Thus, we will review these B cell subsets in TLSs and hope to further understand the function of TLSs. (Fig. 2).

#### Plasma cell

HPV-specific B cells and HPV-specific IgG antibodies were observed in HPV^+^ head and neck squamous cell carcinoma (HNSCC), especially in the B-cell-rich TLS^high^ HNSCC.^57,58^ Notably, peripheral blood lacks HPV-E antigen-specific IgG^+^CD27^+^CD38^+^ PCs, suggesting the humoral immune response is localized rather than systemic.^58^ Germain et al. also reported that PCs produced high levels of IgG and IgA, such as LAGE-1, MAGE family antigens, P53, and NY-ESO-1.^59^ Consistent with HPV^+^ HNSCC, immunoglobulin-producing PCs arise from B cells after being activated by antigen within TLSs in non-small cell lung cancer (NSCLC), rather than from peripheral lymphatic organs.^59^ Moreover, in high-grade serous ovarian cancer (HGSOC) patients, IgG recognizes tumor-associated antigens. The level of IgG3 significantly increases and the level of IgG4 decreases post-chemotherapy in stage 3/4 tumors.^60^ Additionally, IgM, IgE, and IgD deposit in TME.^60,61^ Besides, in the transplanted heart with TLSs, IgM- and IgG-producing PCs increase the possibility of coronary artery damage.^62^ It was also observed in Sjögren syndrome.^63^ Importantly, CD138^+^ PCs around the follicle produce immune reactivity to citrullinated antigens (an antigen determinant for rheumatoid arthritis) by secreting ACPA IgG. Human AID^+^GC^+^ synovial tissue generated ACPA IgG after it was transplanted into the RA/severe combined immunodeficiency chimera model,^64^ indicating that TLSs support antibody production independently of B cells in the peripheral lymph circulation.

#### Regulatory B cell

Breg cells produce TGF-β and/or IL-10, inhibiting the immune response.^65^ While there is currently insufficient evidence to support the existence of Breg cells within TLSs. Given the crosstalk between immune cells and the pro-tumor role of TLSs, we propose that Breg cells may not enter TLSs internal but rather surround them to regulate the formation and function of TLSs. In melanoma, they express pro-inflammatory cytokines, such as TNF-α and IL-6, which are linked to poor clinical outcomes in patients receiving Ipilimumab treatment.^66^ To support immunosuppressive TEM, Breg cells stimulate regulatory T (Treg) cell differentiation, leading to CD8^+^ T cell exhaustion.^67^ In ovarian cancer patients, IL-10^+^ Breg cells preferentially enrich in ascites, which are positively correlated with CD4^+^FOXP3^+^ T cell frequency and negatively correlated with IFN-γ^+^CD8^+^ T cell frequency.^68^ They also generated TGF-β polarizing CD4^+^ T cells into Treg cells.^69^ Additionally, Breg cells also secrete IL-35, directly suppressing CD8^+^ T cells.^70^ Notably, Breg cells regulate Treg cell function by intercellular contact, decreasing the proliferation of CD4^+^ T cells while reducing forkhead box protein P3 (FOXP3) and cytotoxic T lymphocyte-associated antigen 4 (CTLA4) expression in Treg cells.^71^ Moreover, TGF-β stimulates macrophages to shift towards the immunosuppressed M2 phenotype,^72^ or induces dendritic cells (DCs) to overexpress IL-4 and decrease IL-12, altering the Th1/Th2 balance.^73^ In non-cancerous diseases, such as organ transplantation, autoimmune diseases, and skin healing, Breg cells are often associated with immune tolerance by inflammatory molecules, such as IL-10, IL-35, and TGF-β.^74^ Immunotolerant patients typically exhibit a decrease in PCs and specific antibody titers, along with an increase in immature B cells and IL-10^+^ Breg cells.^75^ In allogeneic kidney grafts, the expression of Breg cell markers such as CD5, CD24a, CD38, Cr2, Fcer2a, IL-10, and Havcr1 increased.^76^ Nevertheless, since precise markers of Breg cells are still up for debate, their detection and role in TLSs remain to be further studied.

#### Memory B cell

Multiple memory B cell phenotypes have been detected in TLSs.^6^ Such as IgG1^+^ memory B lymphocytes were enriched in pancreatic cancer, showing a positive correlation with TLS density.^77^ Similarly, a group of memory B cells, including IgG^+^ switched memory B cells (IgG^+^CD38^-^IgD^-^CD27^+^), IgG^-^ switched memory B cells (IgG^-^CD38^-^IgD^-^CD27^+^), and activated switched memory (CD38^-^IgD^-^CD27^+^CD21^-^), was identified in HNSCC.^16^ Notably, both unswitched and switched CD19^+^CD27^+^IgD^+/-^ memory B cells were detected in TLSs and in peripheral blood,^6^ contradicting the previous report that B cell immune response is limited to TLSs. Additionally, it is reported that TLS density and CD27^+^CD19^+^ memory B cell numbers increase after ICB treatment. IgM^+^ memory B cells serve as indicators of patient response to Nivolumab monotherapy in melanoma.^78^ In breast cancer, the atlas of infiltrated B cells revealed a higher proportion of B cells within tumor-associated TLSs, predominantly memory B cells, compared to the naive B cells in peripheral blood. Additionally, TLS contains a greater proportion of B cell clones in TLSs, as well as somatic hypermutation (SHM). Moreover, memory B cells and PCs share VDJ sequences, indicating a common clonal origin.^61^

Although detecting memory B cells in TLSs remains challenging, Sdc1^+^Ighg^+^ memory B cells were also observed in kidney transplantation. They result in immune downregulation, possibly due to their failure to develop into plasma cells.^76^ Moreover, memory B cells were found in close proximity to CD8^+^ T cells in TLS adjacent tumors,^79,80^ which raises a question about the antigen-presenting ability of B cells. Wennhold et al. reported that CD21^-^CD86^+^ B cells accumulated in many tumors with TLSs, including HNSCC, NSCLC, hepatocellular carcinoma (HCC), renal cell carcinoma (RCC), esophagogastric adenocarcinoma, testicular germ cell carcinoma, ovarian cancer, urothelial carcinoma, and colorectal cancer, demonstrating a significant correlation between CD8^+^ T cells and CD21^-^CD86^+^ antigen-presenting B cells.^81^ CD21^−^CD86^+^ B cells trigger an IFN-γ response in CD3^+^ T cells in in vitro studies.^81^ Furthermore, non-typical CD27^−^ memory B cells expressed APC markers, such as major histocompatibility complex (MHC) I, MHC-II, CD40, CD80, and CD86, and colocalized with CD8^+^ T cells in TLSs.^82^ Similarly, CD21^low^CD86^+^IgD^+^CD27^+^ memory B cell subsets highly express MHC-I and MHC-II in rheumatoid arthritis, indicating a robust antigen presentation capacity.^83^

#### Naive B cell

Naive B cells are notably scarce in certain types of tumors, such as breast cancer,^84^ NSCLC,^85^ and melanoma.^86^ Nonetheless, the presence of CD38^−^IgD^+^CD27^−^ naive B cells is related to the GC response within TLSs.^16^ Currently, it remains unclear why there is a difference in naive B cells within TLSs across various tumors, necessitating exploration of naive B cell function and development within TLSs. For example, IgD^+^ naive B cells are reduced in pancreatic cancer, with an increase in mature IgM^+^ B cells and IgG1^+^ memory B cells.^77^ This suggests that naive B cells in tumor sites undergo isotype class switching after antigenic stimulation.

### T lymphocyte subsets

It has been discovered that T lymphocyte subsets correlate with TLSs in many diseases,^87^ such as tumors, autoimmune disorders, infections, and organ transplantation.^17^ However, the presence of T lymphocyte subset infiltration does not always indicate a good prognosis. Here, we will review the T cells within TLSs, hoping for a further understanding of the function of TLSs. (Fig. 2).

#### Tissue-resident memory T cell and exhausted T cell

Tissue-resident memory T (TRM) cells are a special T cell subset. They are perpetually present in peripheral immune tissue and do not participate in lymphatic circulation.^88^ TRM cells and TLSs are associated with a favorable prognosis for tumor patients.^89^ They express the typical marker CD103 and the activating marker CD69, but not CCR7, CD62L, or S1PR1, which are required for migration out of tissue.^90^ In stage III lung adenocarcinoma, the proportion of TRM cells was higher within TLSs, especially in mature TLSs, which is positively correlated with patient survival.^91^ CD103^+^ T cells penetrated the epithelial regions, tumor stroma, and TLSs in gastric cancer tissues. Even in patients with advanced gastric cancer, the infiltration of CD103^+^ TRM cells was also associated with a good prognosis.^89^ Moreover, tumors also contain non-tumor-specific TRM cells, which activate the immune system through the bystander effect.^92^ Nonetheless, TRM cells in progressive tumors frequently indicate immunosuppressive TME since there is an overlap between exhausted T cells and TRM cells in TLSs.^93^ TCR data analysis in NSCLC reveals that CD103^+^CD8^+^ TRM cells express exhausted markers, such as programmed death 1 (PD-1), T cell immunoglobulin domain and mucin domain-3 (TIM-3), and CD39.^94,95^ In addition to the inhibitory molecules, CD103^+^ T cells also express granzyme (GZM) B.^96^ This suggests that TRM cells serve as the source of exhausted T cells, transitioning from an activated state to terminally exhausted T cells without completely losing their function. Compared to CD103^-^CD8^+^ T cells, CD103^+^CD8^+^ T cells express higher levels of PD-1, GZMB, and IFN-γ in TLSs.^89^ Consequently, they are a predictive marker for the effectiveness of ICB treatment in gastric cancer.^97^

CD103^+^ exhausted T cells appear to be the source of CXCL13, a crucial chemokine in TLS formation.^98^ In epithelial ovarian cancer, CD8/CD103/TIM3-expressing lymphocytes secreted CXCL13,^99^ and the density of CD39^+^CD103^+^ T cells with high CXCL13 was linked to better relapse-free survival (RFS) at five years.^100^ CD103^+^ T cells mediate B cell recruitment and TLS formation by CXCL13 under the regulation of TGF-β.^96^ Transcription factor 7^+^ (TCF7^+^) T cells are located in TLSs, and their abundance correlates with a favorable prognosis in oral squamous cell carcinoma (OSCC).^101,102^ Recent studies show that over 80% of CD8^+^ T cells in mouse models of melanoma, lung cancer, colon cancer, and prostate adenocarcinoma express PD-1, which contains TCF1^+^ T cells.^103^ The transcription factor T cell factor 1 (TCF1, encoded by TCF7) contributes to memory T cell differentiation by the Wnt signaling pathway and response to ICB therapy,^104^ alleviating the exhausted state of T cells.^105^ Moreover, TCF1^+^ cells exhibit stem cell-like characteristics similar to memory T cells and are close to the blood vessel.^106^ In autoimmune vasculitis, TCF1^+^CD4^+^ T cell populations are present in perivascular TLSs and exhibit a high proliferative potential.^107^ In chronically lymphocytic choriomeningitis virus (LCMV)-infected mice, TCF1^+^PD-1^+^ T cells infiltrated more intensely and exhibited stem cell-like and CXCR5 expression.^108^ Siddiqui I et al. confirmed that tumor-specific TCF1^+^PD-1^+^CD8^+^ T cells promoted the ICB therapy after applying FTY720 to block the influx of peripheral T cells.^109^

#### Follicular helper T cell

Follicular helper T (Tfh) cells are situated in the B-cell region of TLSs.^110^ Differentiation of Tfh cell is distinct from other CD4^+^ T helper T subsets, which is initiated by the co-stimulatory molecules. Inducible co-stimulator (ICOS) inhibits Klf2 to increase BCL6 expression, promoting Tfh cell differentiation.^111^ CD28 upregulates extracellular regulated protein kinases 2 (ERK2) to suppress BCL6, but this suppression is counteracted by Zfp831, activated by ICOS.^112^ Moreover, TGF-β suppressed SATB1 expression through ICOS de-repression, reducing T follicular regulatory (Tfr) cells and triggering TLS formation.^113^ B lymphocyte-induced maturation protein-1 (Blimp1), an important transcriptional repressor, mediates the differentiation of immune cells.^114^ It drives naive CD4^+^ T cells to turn into CD4^+^ T help cells, excluding Tfh cells.^115^ BCL6 and Blimp1 are inter-antagonistic; the repressive activity of Blimp1 is antagonized by BCL6 (via repressing Prdm1, the gene encoding Blimp1), promoting the differentiation of Tfh cells.^116,117^ Additionally, ICOS promotes nuclear NFAT2 translocation and increases CXCR5 expression,^118^ while CXCL13 attracts CXCR5^+^ Tfh cells to migrate to GC.^119^ Overacre-Delgoffe et al. reported that intestinal microbes suppressed the progression of tumors through Tfh cells, driving TLS development. The colonization of Helicobacter hepaticus increased immune cell infiltration, especially CD4^+^ Tfh cells with TLS formation.^120^ Tfh cells produce cytokines, such as IL-4 and IL-21, promoting the GC response, immunoglobulin switch, and somatic hypermutation (SHM).^121–123^ Besides, cytotoxic Tfh cells express GZMK within TLSs in IgG-4-related diseases, indicating their direct involvement in anti-tumor effects.^124^ In contrast, a recent study showed that the number of TLSs in stage II colorectal cancer (CRC) was less than in stage III CRC. The density of Tfh cells was higher in patients with disease recurrence than in those without recurrence.^125^ Moreover, increased infiltration of Tfh cells after ICB therapy in tumors supports the notion that Tfh “dysfunction” may be attributed to PD-1 expression.^14,126–128^

#### Regulatory T cell

Treg cells are characterized by high expression of CD25 and the transcription factor FOXP3.^129^ They inhibit T cell activation by producing inhibitory cytokines, including IL-10, IL-35, and TGF-β, or by expressing CTLA4 that competes with CD28 to combine with CD80/86.^130–132^ In mouse lung cancer models, Treg cells increased in TLSs around blood vessels and are associated with TLSs in tumor stroma, leading to a poor prognosis for patients. In support of this, the proliferation of CD4^+^ T cells and CD8^+^ T cells was reactivated in TLSs after deleting Treg cells by diphtheria toxin, causing enhanced tumor destruction.^133^ In a mouse lung adenocarcinoma model, the depletion of Treg cells increases CD4^+^ T cell and CD8^+^ T cell infiltration, especially in TLSs. This also increases the co-stimulatory molecule levels of DCs in TLSs.^134,135^ Additionally, Treg cells appear to promote TLS formation, although the mechanism may be indirect.^136^ In the skin TLSs of patients with pemphigus, Treg cells directly contact Th1-like CD4^+^ T cells, which promotes CXCL13 production.^137^

Tfr cells, a unique subset of Treg, have a dual phenotype of Treg cells and Tfh cells. They express FOXP3 as well as BCL6 and the chemokine receptor CXCR5, allowing them to migrate to the GC.^138^ Based on TCR sequencing, Tfr cells and Treg cells are related in development, meaning that Tfr cells may originate from Treg cells.^139^ The differentiation process of Tfr cells highly overlaps with that of Tfh cells and also relies on BCL6 expression, which is regulated by CD28 and ICOS.^111,140,141^ However, compared to Tfh cells, Tfr cells express BCL6 and Blimp1 simultaneously. Hence, Tfr cell differentiation might result from a balance between two opposing-effect cytokines, as their quantity and function are influenced by BCL6 or Blimp1 ablation.^142,143^ In breast cancer, Tfr cells inhibit B cell proliferation and antibody generation by IL-10.^144^ The number of CXCR5^+^FOXP3^+^ Tfr cells in the peripheral blood was higher in early-stage BC patients than in advanced-stage, implicating their involvement in tumor progression and invasion.^145^ Moreover, Tfr cells exhibit a low response to anti-PD-1 therapy. Conversely, anti-CTLA4 therapy or anti-PD-1 therapy after treatment with anti-CTLA-4 achieves desirable therapeutic results,^139^ possibly due to their high expression of CTLA-4.^146^

#### Th17 cell

Th17 cells constitute a crucial component of the T-cell subset in TLSs, and their cytokines contribute to TLS development in chronic inflammatory tissues.^147^ Th17 cells express IL-17 in the nascent lymphoid tissues of renal grafts, promoting immune rejection.^148^ IL-17A also induces iBALT formation in mice with lipopolysaccharide-induced pneumonia.^149^ While IL-22, another effector cytokine expressed by Th17 cells, lacks direct evidence for TLS formation. In IL-23R^-/-^ T cells and Rag1^-/-^ mice, IL-23 drives T cell proliferation and Th17 cell accumulation.^150^ Notably, since naive CD4^+^ T cells lack the IL-23 receptor, IL-23 does not seem to drive Th17 development, while it promotes the stability and survival of Th17 cells.^151^ In esophageal squamous cell carcinoma (ESCC), patients with a promising prognosis had mature TLSs, which are characterized by a high ratio of proliferative B cells and CD4^+^ T cells, including memory B cells and Th17 cells.^152^ However, Th17 cells have also been implicated in facilitating breast cancer progression.^153^ Considering the diversity of Th17 cell functions, their differentiation requires further exploration in order to precisely characterize Th17 cell function. Retinoic acid receptor-related orphan receptor γt (RORγt) and signal transducer and activator of transcription-3 (STAT3) both promote the development of Th17 cells.^154^ The pro-inflammatory factors TGF-β and IL-6 activate RORγt, initiating a cascade of their differentiation.^155,156^ Th17 cells and Treg cells also maintain balance in the differentiation process. FOXP3 negatively regulates RORγt, inducing Treg cell differentiation, and down-regulates Th17 cells through STAT6.^157^ However, the role of IL-21 in Th17 cell differentiation remains controversial, as inconsistent effects of IL-21 on Th17 cells have been found in autoimmune encephalomyelitis and Crohn’s disease.^158,159^ We suggest that this is probably because Th17 cell differentiation is regulated by multiple cytokines. More importantly, IL-21 and IL-6 appear to rely on the same signaling pathway to regulate Th17 cell differentiation. Therefore, it is not sufficient to regulate downstream transcription factors within this pathway alone to demonstrate the role of IL-21 itself.

### Macrophage

Macrophages, particularly pro-inflammatory M1 macrophages, promote TLS formation by producing inflammatory cytokines. (Fig. 2) In atherosclerosis, M1 macrophages act as LTi cells, secreting TNF-α and LTα, triggering the expression of the chemokines CCL19, CCL20, and CXCL16, which induce TLSs in the aortic wall.^160^ A week after infection in the mouse model of Chlamydia pneumoniae (CP), M1 macrophages predominated and contributed to the development of iBALT.^161^ And TLS^high^ tumors exhibit a higher prevalence of M1 macrophages, while TLS^low^ tumors tend to have more M0 and M2 macrophages.^162^ The infiltration of M1 macrophages suggested a favorable prognosis. However, M1 macrophages may gradually switch to the M2 macrophages in iBALT. Adoptively transferring M2 macrophages to CP-infected mice fails to observe iBALT.^161^ Moreover, CX3CR1^+^ macrophages were detected in TLSs, promoting B cell infiltration and IgA responses through TGFβ1, CXCL13, and BAFF, presenting antigen during infectious Salmonella colitis.^163^ Recently, Gunnarsdottir FB et al. have identified the CD169^+^ tissue-resident macrophages in TLSs and have also been proven to produce type I IFN in vitro. Type I IFN stimulates monocyte CD169 expression, which inhibits T cells and NK cells through M2 macrophage-like effects. They secrete prostaglandin E2 (PGE2), reactive oxygen species (ROS), and IL-10 and also contribute to the secretion of antibodies and IL-6 by activated B cells.^164^ In breast cancer, CD169, or TLS, is associated with decreased odds of surviving for 5 years.^165^ Furthermore, TIM4 expression in FOLR2^+^ resident macrophages was positively related to prognosis. Notably, TIM4^+^ macrophages in TLS^−^ tumors express IL-10 and TGF-β, promoting immunosuppressive function, while those in TLS^+^ tumors enhance the prognosis of patients.^166^ In addition, the high expression of PD-L1 within TLSs is mainly from macrophages.^167^ Therefore, inhibition of macrophages or treatment with immune checkpoint blockers significantly enhances T cell and B cell infiltration and promotes tumor regression.^168^

### Dendritic cell

DCs are leukocytes derived from bone marrow. They can uptake, process, and present antigens to T cells.^169^ The infiltration of mature dendritic cells (DCs), T cells, and B cells in TLSs correlates with long-term survival in various cancers, such as lung cancer,^170^ breast cancer,^171^ high-grade serous carcinoma (HGSC),^172^ and OSCC.^173^ Marinkovic T et al. revealed that T cells and CD11c^+^ DCs are recruited to the thyroid gland via CXCL21-CCR7, inducing TLSs in an LTβ signaling-dependent manner.^174^ In diffuse gastric cancer, DC-LAMP^+^ DCs are present in TLSs, and CCL19-CCR7 signaling is crucial for DC homeostasis.^175^ Mature DCs penetrated peribronchially and enhanced lymphocyte aggregation on day 4 after applying murine γ-herpesvirus MHV-68, with organized iBALT observed on day 8.^176^ Further study also confirms the role of DC in promoting TLS formation: bone marrow-derived cells were transferred to the lungs after co-cultured with granulocyte-macrophage colony-stimulating factor (GM-CSF), leading to iBALT development;^177^ and deletion of CD11c^+^ DCs in the diphtheria toxin receptor (DTR) transgenic model results in the absence of iBALT.^174^ Furthermore, cDC2 expressed CXCR5 and co-located with Tfh cells in the T-cell zone of TLSs, polarizing naive CD4^+^ T cells to Tfh cells via ICOS signaling in GOLD stage IV chronic obstructive pulmonary disease (COPD).^178^ Meanwhile, DCs are recruited by respiratory bacteria via CCR2 into SIgA-deficient airways, leading to local disruption of the mucosal barrier and lymphocyte accumulation.^179^ Recently, Song-Yang Wu et al. reported that CCL19^+^ DCs acted as LTi cells in TLS formation and promoted ICB therapy in triple negative breast cancer. They were associated with high levels of CXCL13^+^ T cells and enhanced the efficacy of anti-PD-1 therapy.^171^ Furthermore, in HGSOC, DC-LAMP and CD20 displayed a significant correlation, increasing the levels of the immunocytotoxicity markers GZMA and perforin (PRF1) transcripts.^60^ It suggests that they modulate B cell responses. Additionally, plasmacytoid dendritic cells (pDCs) have been identified as a novel component of the T-cell zone of TLSs. In CRC, pDCs expressed IRF7 and were located close to CD8^+^ T cells, suggesting that they may enhance anti-tumor immunity through secretion of type I IFN and stimulation of CD8^+^ T cells.^180^ Interestingly, IRF7^+^ pDCs preferentially reside around CD4^+^ T cells during TLS development. In another study of breast cancer, pDCs inhibited the proliferation of FOXP3^+^ Treg cells by IFN-α, providing evidence for pDCs regular CD4^+^ T cell function.^181^ (Fig. 2).

### Natural killer cell

NK cells do not require antigenic activation, and the receptors expressed on their surface often represent changes in their function. However, the relationship between NK cells and TLSs remains inadequately explored. A recent study on TLSs in OSCC showed that high-grade TLSs coincided with the infiltration of CD57^+^ NK cells.^173^ Furthermore, NKp30^+^ NK cells increased in Sjogren syndrome with TLSs, activating B cells to produce antibodies (IgG, IgM, and IgA).^182^ Circulating NK cells were recruited to salivary glands before TLS appearance, upregulating NKp46 expression and producing granzyme B and IFN-γ.^183^ Additionally, NK cells act as LTi cells and express IL-22 to induce chemokine production (CXCL12, CXCL13) in stromal cells.^184^

### Innate lymphoid cell

Innate lymphoid cells (ILCs) include three main types: ILC1, ILC2, and ILC3. Among these, ILC3 contributes to the formation and/or maintenance of TLSs. The development of ILC3 depends on RORγt and can be considered as LTi cells. Ikeda A et al. noted a correlation between the abundance of NKp44^+^ ILC3 and the density of TLSs in CRC. Their numbers gradually decline as the cancer develops. However, NKp44^+^ ILC3 is reactivated in advanced CRC under exogenous stimulation, leading to the upregulation of LTα, LTβ, and TNF-α.^185^ This indicated that infiltration of NKp44^+^ ILC3 may contribute to a microenvironment rich in lymphocytes. In NSCLC, NCR^+^ ILC3 increases in proximity to TLSs, producing IL-22 in response to IL-23 stimulation.^186^ Another study reported that IL-21 increases IL-22 and Tbet production by ILC3 while decreasing IL-17 expression, thereby exerting a protective role in intestinal inflammation.^187^ Altogether, the available evidence suggests that ILC3 acts as LTi cells involved in TLS formation, which can be activated by diverse stimuli to produce multiple effectors.

### Neutrophil

The ratio of peripheral blood neutrophils and lymphocytes, along with TLSs, was described as a tumor prognostic marker in patients with HCC and uterine leiomyosarcoma.^188–190^ Neutrophils can also be found within TLSs in omentum metastases of HGSOC and prostate cancer.^60,191^ However, their function has not been characterized. A recent study revealed that the number of CD15^+^ neutrophils correlates with TLS density. TLS^-^ tumors exhibited better overall survival and disease-free survival compared to TLS^+^ tumors in peritumoral tissue.^192^ In fact, neutrophils express BAFF, IL-21, IL-17, and MHC, providing activation signals for B cells and T cells.^193–195^ Therefore, we believe that the role of neutrophils in TLSs deserves further investigation.

## Cytokines involved in TLS formation

The cytokines and chemokines have been demonstrated to play a crucial role in the development of TLSs in mouse models. These molecules induce TLSs with distinct characteristics, consequently increasing the number and activity of lymphocytes within the TME (Table 1).

**Table 1:** Overview of cytokines in promoting TLS formation

Cytokine	Type	Donor cells	Recipient cells	Function	Ref.
Interleukins	IL-4	Tfh cells	B cells, FDCs	Inhibiting B cell differential in GC	^226^
	IL-6 family (IL-6, IL-11, OSM, IL-27)	Stromal cells	T cells	Modulate differentiation of Tfh cells and Th17 cells	^196,203,206^
	IL-7	Fibroblasts, FDCs, Macrophages, DCs, ECs	B cells, T cells, EC	Promote LTi cells to secrete LTα1β2	^208,209^
	IL-13	ILCs	Fibroblasts	Activate fibroblasts to form the immune fibroblast network and express chemokines	^41^
	IL-17	Th17 cells	Fibroblasts	Promote fibroblast expansion and expression of chemokines	^149,222^
	IL-21	Tfh cells	B cells	Promote the proliferation, maturation and differentiation of B cells	^113,122^
	IL-22	Th 17 cells, ILCs	Fibroblasts	Expand the fibroblast network and induce the secretion of chemokines	^184,217^
	IL-23	DCs, Macrophages	Th17, ILCs	Induced Th17 cells and ILCs to secrete IL-17 and IL-22	^228,229^
	IL-1 family (IL-1β, IL-36γ)	DCs, Macrophages, ECs	T cells, Stromal cells	Increases the expression of CXCL13, CCL21, LTα, and LIGHT	^198,199,562^
	IL-10	-	Macrophages	Upregulate CXCL13	^227^
Chemokines	CXCL12	Stromal cells	B cells	Recruit B cells and plasma cells; mediate B cell migration from light zone to dark zone in GC	^222,367^
	CXCL13	T cells, B cells, Stromal cells	B cells, Tfh cells	Recruit B cells and Tfh cells, induce B cells to secrete LTα1β2; mediate B cells migrate from dark zone to light zone in GC	^119,254,257^
	CCL19	Fibroblasts, DCs	T cells, DCs	Recruit T cells and DCs to promote the formation of T cell zones	^13,171^
	CCL21	Stromal cells	T cells, DCs	Recruit T cells and DCs; induce T cells express LTα1β2	^262^
	CX3CL1	Stromal cells, B cells	B cells	Recruit B cells	^267^
TNF super family members	Lymphotoxin (LTα1β2, LTα, LTα3)	T cells, B cells, DCs	Fibroblasts, ECs	Induce the expression of chemokines and adhesion molecules; promote the formation of HEVs	^209,251,301^
	LIGHT	T cells, B cells, DCs	ECs	Induce ECs to convert to LECs, inducing formation of HEVs	^238,239^
	TNF-α	T cells, B cells, Macrophages, ILCs	Fibroblasts, ECs	Active fibroblasts; induce fibroblasts express ICAM-1, VCAM-1, CXCL13, CCL19, and CCL20	^233,240^
	BAFF (TNFSF13)	FDCs	B cells	Promote the survival and proliferation of B cells	^51,247^
Interferon	Type I IFNs	-	Fibroblasts, Neutrophils, T cells	Induce CXCL13, BAFF expression; promote Tfh cell differentiation	^268,269,271^
	IFN-γ	T cells	B cells、ECs	activate B cells by upregulating BAFF and IL-6 expression; promote HEV formation	^272,274^
TGF-β	Fibroblasts	T cells, ECs	Promote CXCL13 expression; modulate Tfh cell differentiation; promote HEV formation	^113,276,277^	
Other	VEGF	ILCs	ECs	Enhance EC proliferation and expansion	^281^
	BCL6	Tfh cells, FDCs	Tfh cells, B cell	Promote Tfh cell differentiation and B cell maturation	^287^
	AID	-	B cells	Associated with B-cell isotype switching and SHM	^291,292^
	GM-CSF	-	DCs, Treg cells	Increase mature DC infiltration; inhibit Treg cell proliferation	^293^

### Interleukins

Interleukins are cytokines that mediate interactions between leukocytes and/or lymphocytes, activating and regulating immune cells, playing an important role in inflammatory responses. Furthermore, it acts as a signaling molecule to regulate TLS neogenesis.

#### IL-1 family

The IL-1 family includes 11 members of the cytokines and shares similar functions with Toll-like receptor (TLR) family. IL-1β accelerates the early inflammatory process and initiates kidney-specific TLS formation in lupus nephritis.^196^ In mice infected with the Influenza A virus (IAV), IL-1α administration increases the number of iBALT in the lung. However, IL-1r^−/−^ mice, despite surviving long-term post-IAV infection, show no iBALT formation due to the IL-1 signaling pathway blockade, and CXCL13 expression is also reduced.^197^ Furthermore, an over-three-fold increase in IL-1β and IL-1α was observed on day 7 following intranasal instillation of cSiO2, leading to TLS development after 21 days in the lungs.^198^ Weinstein AM et al. reported that IL-36γ is predominantly expressed by M1 macrophages and endothelial cells (ECs) in colon cancer mouse models, including SMA^+^ smooth muscle cells and PNAd^+^ HEVs. Their expression increases the density of CD4^+^ T cells, CD20^+^ B cells, and PDPN^+^ fibroblasts in TLSs, supporting a favorable prognosis.^199^

#### IL-6 family

The IL-6 family includes IL-6, IL-11, oncostatin-M (OSM), and IL-27.^200^ OSM-expressing adenoviral vectors to activate B cells, leading to iBALT formation in mice’s lungs.^201^ In IL-6 and IL-6R double transgenic mice, peribronchial lymphocyte aggregation was observed, accompanied by the upregulation of CXCL13 and the GC response.^202^ However, IL-6 may indirectly induce TLS formation by promoting Th17 cell differentiation.^203^ In lupus nephritis, IL-6-producing mesenchymal stem cells promote CD4^+^ T cell proliferation and differentiate into Th17 cells in contact form.^196^ B cells also secreted IL-6, enhancing Th17 cell proliferation and polarizing Th17 cells to PDPN^+^ Th17 cells with the collaboration of CD4^+^ Tfh cells.^204^ Additionally, IL-6 was also reported to facilitate Tfh cell differentiation during chronic viral infection.^205^ IL-17 and STAT3 are highly expressed within TLSs in an experimental model of gastric cancer. However, it appears to promote gastric cancer progress.^206^ Notably, IL-27 is the only subtype of the IL-6 family that is reported to inhibit TLS formation by suppressing Th17 differentiation.^207^

#### IL-7

IL-7 plays a crucial role in all TLS development stages, including initiation, expansion, and maturation.^208^ Stromal cells secrete IL-7, which promotes the production of lymphotoxin α1β2 (LTα1β2) by lymphocytes. In viral-induced salivary gland inflammation, IL-7 and LTα1β2 synergistically promoted HEV establishment by inducing vascular endothelial growth factor C (VEGF-C) in stromal cells. IL-7 supports early lymph vascular remodeling, whereas LTα1β2 regulates the complex HEV networks.^209,210^ Notably, the expression of IL-7 significantly increases and precedes lymphatic vessel expansion in submandibular gland inflammation. Lymphatic endothelial cells (LECs) specifically express IL-7Ra, implying that fibroblast-derived IL-7 appears to support HEV development in a paracrine manner prior to LTα1β2.^209,211^ In addition, FDCs supported B cell survival, proliferation, and maturation by secreting IL-7 in GC.^212^ In the T-cell zone of TLSs, fibroblastic reticular cells (FRCs) secrete IL-7 to promote the survival of central memory T cells, thereby maintaining T cell homeostasis.^213^ Thy1 IL-7^+^ LECs generate IL-7, supporting the maintenance of memory Th2 cells in iBALT by inducing antiapoptotic protein BCl2 expression.^214^

#### IL-13 and IL-22

IL-13 is an indispensable cytokine for fibroblast activation, initiating TLS formation. The primary sources of IL-13 and IL-22 include T cells, ILCs, and NK cells.^184,215,216^ Upon stimulation by IL-13, fibroblasts switch to PDPN^+^FAP^+^ immunofibroblasts, accompanied by the upregulation of ICAM-1 and VCAM-1, which increase chemokine production.^41^ While direct evidence linking Th17-derived IL-22 to TLS formation is lacking, research suggests a positive correlation between IL-22^+^ ILC3 and TLSs.^217^ In primary Sjögren’s syndrome (pSS), the introduction of exogenous IL-22 resulted in PDPN^+^ FAP^+^ fibroblast expansion within the pathological parotid gland.^41^ And the function of FRCs obtained from IL-22^−/−^ mice is impaired, affecting chemokine production and showing defects in TLS formation in vivo.^184^

#### IL-17

IL-17 plays a key role in the formation of TLSs.^218^ Th17 cells secrete IL-17, which promotes FRC proliferation, maintains the FRC network, and induces the secretion of CXCL13 and CCL19.^219^ In IgG nephropathy, blocking IL-17A significantly impaired TLS formation, which decreased kidney damage.^219^ It can also be observed that IL-17 promotes TLS formation in the inducible bronchus-associated lymphoid tissue (iBALT).^220^ However, Fleige H. and colleagues found that IL-17 is not a determinant of iBALT development. They successfully induced normal iBALT in IL-17A and IL-17F double-deficient mice with the cowpox virus.^221^ Similarly, cowpox virus-induced organized iBALT was also observed in IL-17-deficient mice.^222^ This suggests that IL-17 may have different functions in various organs and pathological conditions.

#### IL-21

IL-21, produced by Tfh cells, promotes cooperation between Tfh cells and B cells to maintain the GC response.^96^ A recent study demonstrated that IL-21 derived from CD4^+^ T cells directly enhances CXCL13 expression in myeloid cells in ICB therapy-induced immune-related adverse events.^223^ TGF-β mediated silencing of SATB1 resulted in Tfh cell differentiation and IL-21 expression, thereby driving TLS development, which is critical for ovarian tumor control.^113^ Within TLSs of transplanted kidneys, IL-21 is located in the GC, activating B cells and leading to graft failure.^224^ Moreover, Tfh cells secreting IL-21 in TLSs promote renal fibrosis in chronic kidney disease, and treatment with ICOS antibodies alleviates disease progression.^225^

#### Others

Apart from the interleukins mentioned previously, others also influence the formation of TLSs, although the specific mechanisms remain incompletely understood. For example, IL-4 from Tfh cells activates IL-4R on CD23^+^ light zone (LZ) GC-B cells, leading to STAT6 phosphorylation and inhibiting memory B cell development. Whereas IL-4R^+^ FDC binds IL-4 competitively to relieve its suppression of B cell differentiation.^226^ IL-21 appears to play a crucial role in this process.^121^ Additionally, IL-10 upregulates CXCL13 in lung macrophages and monocyte-derived macrophages through activation of the JAK/STAT pathway.^227^ However, whether it is associated with TLS development remains unclear and requires further investigation. Besides, IL-23 can induce IL-17 and IL-22 expression by Th17 cells,^228^ and promote the generation of IL-22 by ILCs and γδ-T cells.^229^

### TNF-superfamily members

Tumor necrosis factors, a type of pro-inflammatory cytokine, can kill tumor cells or cause necrosis of tumor tissue through their cytotoxicity. TLS development has been shown to be dependent on cytokines from the TNF family, particularly lymphotoxin, TNF-α, and LIGHT.

#### Lymphotoxin

Lymphotoxin, a member of the TNF superfamily, is the key to lymphoid tissue development and exists in two main forms: lymphotoxin α (LTα) and lymphotoxin β (LTβ). Activated lymphocytes produce soluble homotrimers (lymphotoxin α3, LTα3) formed by LTα that are also known as TNF-β. LTα forms a transmembrane heterotrimer (lymphotoxin α1β2, LTα1β2) on the cell surface when it interacts with LTβ through their ectodomains. LTβR, also recognized as TNF receptor superfamily member 3, is expressed only by stromal cells and myeloid cells, not lymphocytes.^230,231^ This distribution is significant since it underscores the specificity of cellular interactions. The interaction between LTα1β2 and LTβR serves as a communication signal bridging lymphocytes, stromal cells, and myeloid cells through a non-classical NF-κB pathway dependent on the IKK complex.^232^ The LTα1β2 promotes stromal cells to express adhesion molecules and chemokines, such as CXCL12, CXCL13, CCL19, CCL21, VCAM-1, and ICAM-1. Additionally, it has also been reported to upregulate PANd expression in ECs and induce HEV transformation.^233,234^

#### LIGHT

The ligand for LTβR is not limited to LTα1β2; it is also activated by LIGHT, also known as the TNF superfamily 14. LIGHT is primarily expressed in activated T cells and DCs.^235,236^ Herpes virus entry mediator is an additional receptor for LIGHT; however, it appears to have no function in TLS development. The LIGHT-LTβR pathway in TLSs mainly induces HEV formation and promotes lymphocyte migration.^237,238^ Notably, many scholars have identified LIGHT as a potential modulator of tumor immunity. For example, He et al. developed a short peptide, CGKRK-LIGHT, to target tumor vasculature, successfully inducing HEV formation and lymphocyte infiltration in glioblastoma.^239^ CGKRK is a vascular targeting peptide (VTP) that targets specific receptors on tumor vessels. LIGHT-VTP normalizes tumor vasculature, enhances chemokine production by ECs, and promotes TLS formation.^36^ Surprisingly, combining LIGHT and ICB therapy improved the therapeutic efficacy against tumors.^36^

#### TNF-α

Although lymphotoxin signaling is indispensable for the development of TLSs, TNF-α also has a non-negligible role in the absence of lymphotoxin signaling. In particular, LTi cells are not necessary for TLS occurrence. For example, TNF-α activates stromal cells to produce chemokines, driving the formation of gut TLSs in an inflammatory environment lacking RORγt^+^ LTi cells.^240^ Furthermore, it also regulates HEV development independently of LTβ. In melanoma mice, blocking LTβR did not affect HEVs, whereas TNFR1/2^−/−^ mice lacked PNAd^+^ HEVs.^241^ However, TNF-α does not appear to induce mature HEVs, and a more mature HEV phenotype still requires LTβR signaling.^233,242^

#### BAFF

B cell activating factor (BAFF), also known as TNFSF13B (CD257), belongs to the tumor necrosis factor ligand family.^243^ It binds to BAFFR, activating the non-classical NF-κB2 pathway, which increases the expression of the BCL family with anti-apoptotic activity. Moreover, BAFF enhances mitochondrial function and increases ATP production through the PI3K pathway. Both of these pathways improve the survival of B cells. The B cell activation marker CD83 and BAFF co-enriched in the B-cell zone of TLSs in encephalomyelitis.^244^ It suggests that elevated levels of BAFF contribute to B cell activation and PC differentiation.^245^ Conversely, blocking BAFF leads to a decrease in newly formed TLSs, reducing infiltration of B cells and T cells, preventing TLS formation, and mitigating lupus nephritis.^246–249^

### Chemokines

Chemokines are secondary pro-inflammatory mediators induced by primary pro-inflammatory mediators, such as interleukins or TNF.^250^ It promotes immune cell migration and lymphoid tissue neogenesis by binding to specific receptors on the cell surface. Therefore, chemokines are critical for TLS formation and maintenance.

#### CXC subfamily

CXCL13 recruits B cells by selectively binding CXCR5. Initially, activated stromal cells, particularly FRCs, are the main source of CXCL13 during the early stages of TLS development.^251^ Subsequently, macrophages, DCs, FDCs, and T cells also act as lymphoid tissue organizer (LTo) cells and produce CXCL13.^252–255^ Moreover, before GC maturation, fibroblasts and lymphocytes are the primary producers of CXCL13. Notably, recruited B cells also generate CXCL13, which creates a positive feedback loop.^43^ After the constitution of the FDC network, FDCs become the main cells producing CXCL13 in GC.^254^ Tfh cells, in addition to B cells, express CXCR5 and migrate into the TLSs following the CXCL13 gradient, promoting GC maturation.^119^ Therefore, CXCL13 is often used as a marker for TLS formation and tumor prognosis.^256^ However, high levels of CXCL13 do not always indicate a favorable prognosis. Recent studies have shown that infiltration of CD8^+^ T cells with high CXCL13 expression in RCC results in an immunosuppressive microenvironment.^257^ Apart from CXCL13, stromal cells secrete CXCL12, which is also shown to promote B lymphoid follicle formation in the absence of FDCs.^222^ CXCL12/CXCR4 and CXCL13/CXCR5 mediate the migration of B cells between the dark zone (DZ) and light zone (LZ) in the GC. In the presence of CXCL13, GC B cells aggregate in the LZ for antigen selection, whereas CXCL12 attracts them to the DZ to undergo SHM.^258^ Furthermore, although CXCL9 and CXCL10 can attract T cells, their direct involvement in TLSs remains unclear. Nevertheless, they have been shown to enhance antitumor immunity in melanoma and are associated with the expression of TIS-related genes.^259^

#### CC subfamily

CCL19 and CCL21, which bind to CCR7, are primarily produced by stromal cells and ECs, attracting T cells and facilitating the development of a T-cell zone in TLSs.^260,261^ In triple-negative breast cancer, the presence of CCL19^+^ DCs has been linked to TLSs and T cell aggregates. Elevated levels of CCL19 improved ICB therapy outcomes and increased patient survival.^171^ The levels of CCL21 in peripheral blood also positively correlate with the efficacy of ICB therapy, with CCL21 expression observed in TLS-like areas.^262^ In lung cancer patients with a history of smoking, they have higher levels of TLSs and T cell infiltration, which is attributed to tobacco stimulation increasing CCL21 expression. However, CCL21 is not secreted by stromal cells but by lung adenocarcinoma (LUAD) tumor cells.^263^ Additionally, CCL20 has also been found to be upregulated in tumors containing TLSs,^264^ although direct evidence linking CCL20 to TLS formation is still lacking.

#### CX3CL1

CX3CL1 is the only member of the CX3C chemokine family associated with various diseases.^265,266^ Within TLSs, CX3CL1 expression was detected within the B-cell zone, while its receptor, CX3CR1, is predominantly found in the CD3^+^ T cell region.^267^

### Interferons

Type I IFN stimulated PDGFRα^+^ fibroblasts to secrete CXCL13, which attracted naive B cells and initiated TLS development. It is worth noting that this process still required Tfh cell assistance.^268^ Additionally, type I IFN increased BAFF expression in neutrophils through the transcription factors IRF1 and IRF2,^269^ which might contribute to B cell proliferation and GC formation within TLSs. Andersson A et al. reported that the type I IFN pathway is enriched and co-localized with CXCL10^+^ macrophages in HER2-positive breast cancer. They also observed a connection between type I IFN-associated M2 macrophages and T cells in TLSs. However, they did not detect the GCs, implying TLSs may not be fully mature yet.^270^ Furthermore, type I IFN also directly induced STAT1 binding to BCL6, promoting CD4^+^ T cell differentiation into CXCR5^+^ PD-1^+^ Tfh cells.^271^ IFN-γ, a type II IFN, indirectly induces HEV formation by increasing lymphocyte infiltration.^272^ IFN-γ secreted by Th1 cells increases chemokine production in high-grade muscle-invasive bladder cancer, promoting TLS formation and enhancing the sensitivity to treatment.^273^ Additionally, it may also activate B cells, triggering TLS formation by upregulating BAFF and IL-6 expression.^274^

### Transforming growth factor-β

TGF-β is an immunosuppressive factor that regulates immune homeostasis and tolerance, facilitating tumors in evading the immune system and resisting cancer immunotherapy.^275^ Several studies have shown an upregulation of TGF-β expression within TLSs. For instance, in CnAα^+/-^ hybrid mice, LYVE-1^+^ ECs have been found to promote the spontaneous formation of TLSs. TGF-β could potentially create a conducive environment for the transformation of ECs into HEVs and the formation of TLSs.^276^ Additionally, TGF-β inhibits SATB1 activity, leading to the differentiation of CD4^+^ T cells into Tfh cells that express LIGHT, CXCL13, and IL-21.^113^ In pancreatic ductal adenocarcinoma (PDAC), TGF-β derived from fibroblasts activates T cells, stimulating them to secrete CXCL13.^277^ Moreover, another study has demonstrated that TGF-β activated T cells, resulting in their differentiation into CXCR5^-^BCL6^-^ peripheral Th (Tph) cells that express CXCL13. It also reduces IL-2 production, driving CXCL13 upregulation in CD8^+^ T cells through the expansion of Treg cells.^278^ Moreover, in pemphigus chronic blisters, Treg cells have been shown to increase CXCL13 production in CD4^+^ T cells by mediating the attenuated stimulation of TGF-β in TLSs.^137^

### Other

Vascular endothelial growth factor (VEGF) levels are elevated in various diseases, such as cancer and blinding eye diseases.^279^ It promotes tumor angiogenesis and regulates lymphatic vascularization.^279^ LTβ signaling stimulates FRCs to express VEGF, which enhances EC proliferation and expansion; notably, VEGF had a greater impact on PNAd^−^ ECs.^280^ NRP1^+^ ILC3 located near the HEVs produce VEGF-A and act as LTi cells, promoting TLS formation in COPD.^281^ Additionally, anti-VEGF/VEGFR2 treatment has been found to convert tumor blood vessels into HEVs, enhancing T-cell permeation.^282^ B cell lymphoma 6 (BCL6) is essential for B cell maturation during the GC response, and BCL6 expression is specifically regulated.^283^ It targets Myc and Prdm1, inhibiting the processes of positive selection and differentiation of PCs, respectively.^284^ The mice lacking BCL6 fail to form GC.^285^ In addition, it also promotes Tfh cell differentiation.^286^ A recent study has shown that BCL6 regulates the interaction between Tfh cells and B cells to maintain the GC response.^287^ Notably, BCL6^+^ GC formation is a hallmark of mature TLSs.^288,289^ Activation-induced cytidine deaminase (AID) is associated with B-cell isotype switching and SHM, promoting the production of high-affinity antibodies in TLSs.^290^ In uveitis, Epps et al. observed that TLSs have distinct B cell and T cell zones, with expression of AID and BCL6 in the GC.^291^ Increased expression of AID leads to the differentiation of B cells in pemphigus skin lesions.^292^ And it is also often used as a marker for mature GCs. The intra-tumoral TLSs present in PDAC patients treated with the GM-CSF-secreting allogeneic PDAC vaccine (GVAX) led to the infiltration of CD83^+^DC-LAMP^+^ DC and the inhibition of Treg cell proliferation.^293^

## Cascade regulatory mechanisms of TLS formation

### Enlightenment of SLO development for TLS formation

TLSs lack some structures compared to secondary lymphoid organs (SLOs). The development of SLOs is a complex and ordered series of processes that occur in specific anatomical locations during the embryo, whereas the appearance of TLSs is a postnatal response to persistent inflammatory signals. Although TLSs are acquired and lack a specific site, their formation also involves interactions between lymphocytes and non-lymphoid stromal cells.^234,235^

TLSs are similar to SLOs in structure and function. Therefore, a thorough understanding of SLO development will enhance our comprehension of TLS formation. SLOs, encompassing lymph nodes, spleen, tonsils, Peyer’s patches, and mucosa-associated lymphoid tissues, are distributed throughout the body.^294^ The formation of SLOs is tissue-specific, involving a complex interplay between hematopoietic cells and non-lymphoid stromal cells in the embryonic stage.^2^ LTi cells, derived from fetal liver-generated IL-7Rα^+^Sca-1^low^c-Kit^low^ progenitor cells, colonize around E12.5-E14.5 and express RORγt and ID2, representing ILCs. They migrate to lymph node progenitors through the chemokine receptors CXCR5 and CCR7.^295^ LTi cells express and deliver LTα1β2, which binds to the LTβR on LTo cells, promoting up-regulation of adhesion molecules such as VCAM-1, ICAM-1, and MADCAM-1. Additionally, this interaction increases the secretion of homeostatic chemokines, such as CCL19, CCL21, and CXCL13.^294^ Subsequently, these molecules collaborate to regulate immune cell recruitment to lymphoid niches, facilitating adult lymphoid structure development. Silencing LTα, LTβ, and LTβR resulted in lymphatic node (LN) defects.^296^ Furthermore, IL-7 triggers a positive feedback loop, inducing the secretion of LTα1β2 by LTi cells.^294^ Long-term interaction between LTi cells and LTo cells induces lymphoid tissue development, promoting the selective entry of naive T and B cells into lymph nodes.^297^

**Fig. 3:**
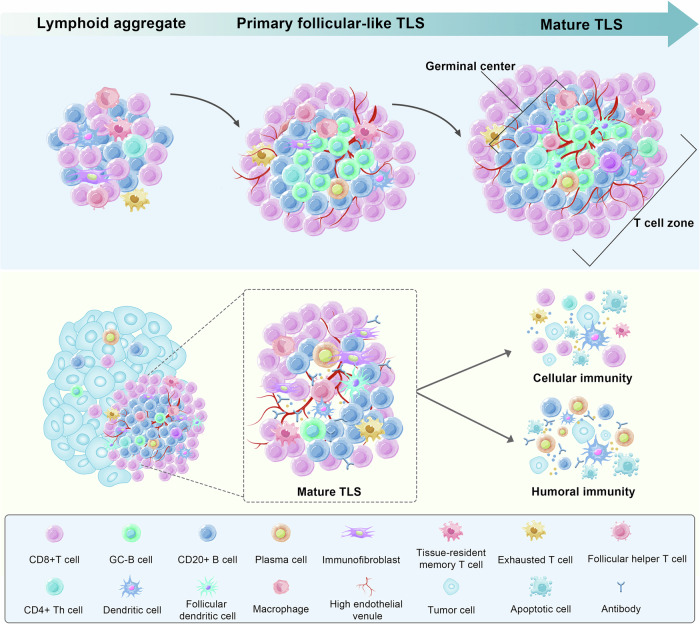
The development stage and structure of TLS. The diagram illustrates the presence of TLSs in tissue. Firstly, dense lymphoid aggregates infiltrate the tumor tissue. Subsequently, primary follicular-like TLSs show B-cell zone and T-cell zone, but lack GCs. Mature TLSs are characterized by mature GCs and the presence of FDCs. In TLSs, CD8 + T cells have cytotoxic effects, and plasma cells produce antibodies, which promote tumor regression

In general, the development of TLSs is divided into three stages. The first stage consists of dense lymphocyte aggregates with a large number of fibroblasts. However, there are no separate T-cell and B-cell zones, nor FDCs. It can be considered a “precursor” to TLSs. The second stage is referred to as “early” or “primary follicular-like” TLSs, in which T cells and B cells gradually form distinct areas, but there is a lack of GC response. The third stage is considered “mature” TLSs, which are characterized by a well-organized T-cell zone and B-cell zone and the presence of an FDC network in GCs (Fig. 3). In addition, HEV development occurs throughout TLS formation. Recently, Liu et al. reported the developmental process of TLSs in LUAD. They mapped the immune landscape during the development of LUAD by scRNA-seq on immune cells in the initiation, invasion, and progression stages of LUAD. In the early LUAD, CD4^+^ T cells prime aggregated around alveolar epithelial cells, and CD8^+^ T cells and B cells were scattered, constituting TLS precursors; with tumor invasion, Tfh cells appeared and were accompanied by B cell aggregation, forming early TLSs; finally, mature B cell zone and T cell zone was formed, which were defined as mature TLSs. In addition, the mature TLSs contained GC, PNAd^+^ HEVs, and CD21^+^ FDCs. More importantly, as the tumor progressed, the TLS density and the ratio of TLSs to tumor area increased. This may indicate that TLS mature with tumor progression and is positively correlated with better patient survival.^298^ Furthermore, it has also found three stages of TLS development in gastric cancer: secondary follicle-like TLSs, primary follicle-like TLSs, and early TLSs, which contained DCs and PNAd^+^ HEVs.^299^

### Priming of stromal cells as a crucial step in the formation of TLSs

#### The origin of signals for activating stromal cells

**Fig. 4:**
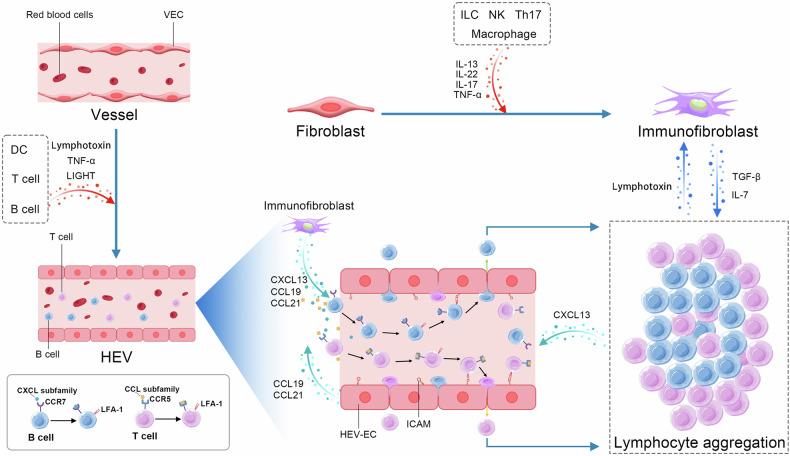
Stroma cells initiate the formation of TLS. The sketch displayed that activation of fibroblasts and vessel endothelial cells (VECs) initiates the formation of TLS, leading to the production of chemokines and promoting the migration of lymphocytes. During this process, IL-7 mediates the positive feedback loop between the stroma cells and lymphocytes. HEVs promote the transport of lymphocytes. Chemokines stimulate the expression of LFA-1 on lymphocytes. It binds to adhesion molecules (ICAM) and stops the rolling of lymphocytes. Subsequently, they cross over the HEVs and are recruited to specific regions formed by fibroblasts

As early as 2004, Cupedo et al. injected subcutaneously CD45^+^CD4^+^CD3^−^ cells from LN and induced lymphoid neoorganogenesis in vivo after 2 weeks, emphasizing the critical role of stromal cells in TLS development.^300^ The priming of stromal cells, especially tissue fibroblasts, forms a fibrous reticulum scaffold, providing space for TLS formation.^43^ (Fig. 4) Despite controversies surrounding the cellular and molecular requirements for stromal cell differentiation and specialization during TLS establishment, IL-13, IL-22, and IL-17 are still acknowledged as pivotal factors. The activation of “immunofibroblasts” relies on IL-13 signaling, where IL-13 binds to IL-4R rather than IL-4.^41^ IL-17 and IL-22 promote their further proliferation.^149,184^ Furthermore, fibroblasts specifically bind to LTα1β2 and LIGHT by LTβR, stimulating their proliferation.^251^ LTα3 also binds to LTβR and mediates TLS development, which depends on the upregulation of ICOS-L by immune fibroblasts and other APCs.^301^ And LTβR deficiency impedes TLS formation.^302^ Furthermore, myeloid cells also induce stromal cells to secrete chemokines by TNF-superfamily members and TGF-β, which trigger lymphoid cell recruitment into TLSs.^160,185,303^

In different organs, the stromal cells that trigger TLS formation appear tissue-specific. For example, TLSs always accumulate around alveolar epithelium cells or bronchi in lung cancer. More importantly, CD4^+^ T cells preferentially accumulate around the pulmonary alveolar epithelium cells in LUAD and subsequently initiate TLS development.^298^ Another study showed the activation of lung resident stromal cells in transplanted lung, including airway epithelial cells and type II pneumocyte-like cells. They produce CXCL12 and recruit lymphocytes.^304^ In addition, kidney-resident stromal cells promote TLS formation in lupus nephritis, such as podocytes, mesangial cells, and renal tubular epithelial cells.^305^ In lupus nephritis, nucleosome-containing immune complexes in the mesangial matrix lead to mesangial cell activation, inducing a high degree of chemokines, including CCL2, CCL7, CCL20, CXCL1, CXCL2, and CXCL5. This induces an infiltration of neutrophils, macrophages, T cells, and B cells in the renal stroma.^306^ Renal tubular epithelial cells express IL-23 receptors, increasing intracellular calcium flux, upregulating glycolysis, and enhancing L-arginine levels in response to IL-23, which regulate T cell proliferation and function.^307^ In addition, renal tubular epithelial cells also express BAFF and IL-6.^308,309^ These evidence suggest that renal stromal cells may play an important role in the development of TLSs in renal diseases

#### Activated stroma cells assumed the role of LTo cells

Activated stromal cells, acting as LTo cells, raise CD3^+^ T cells and B220^+^ B cells to TLSs by secreting chemokines such as CXCL12, CXCL13, CCL19, and CCL21, which bind to CXCR5 and CCR7, respectively.^43,251^ The transcription of CXCL13, CCL19, and CCL21 mRNA significantly increased prior to TLS development and peaked when TLSs were fully developed.^310^ In mice with melanoma, fibroblasts express high levels of CXCL12 and CXCL13, leading to the aggregation of T cells and B cells in the PDPN^+^ network and inducing follicle formation in TLSs.^43^ And CXCL13 inhibitors depress TLS formation.^302^ Notably, IL-17-induced CXCL12 recruits B cells and induces follicle formation in the absence of CXCL13^+^ FDCs.^222^

#### A positive feedback mechanism during TLS formation

Fibroblasts secrete chemokines to attract T cells and B cells for infiltration. In turn, T cells and B cells express several cytokines to promote fibroblast activation and proliferation. This positive feedback mechanism significantly improves the efficiency of TLS formation. (Fig. 4) Migrating lymphocytes accumulate in the early TLS stages and produce CXCL13. A recent study found that B cells recruited by fibroblasts produce more CXCL13 than fibroblasts themselves, facilitating the expansion of the lymphoid aggregates. This process appears to equally depend on LTβR signaling.^302^ In mice lacking lymphocytes or B cells, the CD45^+^PNPD^+^ network is reduced, along with poor TLS size and number.^43^ However, depleted B cells did not affect TLS function in multiple sclerosis.^311^ CXCL13^+^ T cells are pivotal in recruiting CXCR5^+^ B cells in TLSs and are associated with a favorable prognosis.^312^ TGF-β interacts with TCR in a chronic stimulation form, promoting CXCL13 expression by CD39^+^CD103^+^ TRM cells.^257,313^ In HGSCs with TLSs, CD4^+^ T cells and CD8^+^ T cells are the main sources of CXCL13, but as TLSs mature, CXCL13 is gradually expressed by CD21^+^ FDCs.^254^ Furthermore, fibroblasts produce IL-7, which supports LTi cells expressing LTα1β2 in TLSs, and IL-7^-/-^ mice observe abnormal lymphoid organ development.^208^ Luther SA and colleagues reported that LTα1β2 expression in LTi cells depends on IL-7Rα, and their recruitment in vivo was mediated by CXCL13^+^ stromal cells.^314^ Furthermore, lymphocytes recruited by chemokines secrete LTα1β2, which binds to LTβR, inducing fibroblast proliferation and maturation.^10^

### Critical role of HEVs in TLS formation

HEVs originate from postcapillary venules and express PNAd to bind L-selectin on lymphocytes, enhancing lymphocyte migration into TLSs. HEVs have been detected during the early stages of TLS development,^315^ and they are associated with survival.^316^ (Fig. 4)

#### The occurrence of HEV

To alleviate hypoxia during rapid tumor progression, tumor cells secrete hypoxia-inducible factor, VEGF, and platelet-derived growth factor to stimulate tumor endothelial cells (TECs), fostering angiogenesis. Tumor vessels are characterized by an incomplete wall, a lack of smooth muscle, and incomplete pericyte coverage. They restore integrity under the stimulation of inflammatory cytokines and convert to HEVs in TLS formation. Vascular remodeling recruits lymphocytes to inflammatory tissues and coordinates TLS formation in chronic inflammatory conditions.^317^ TLS-HEVs exhibit a hybrid phenotype of TECs and LN-HEVs, suggesting that they may originate from TECs and share similar functions to LN-HEVs.^13^ HEVs, function as lymphatic vessels, are specialized post capillary microvessels, serving as the primary channel for lymphocyte trafficking within TLSs,^242^ with their formation reliant on persistent LTβR signaling.^13,318^ In Sjogren’s syndrome, lymphatic aggregates are characterized by a gp38^+^/pdpn^+^ fibroblast network, accompanied by CD31^+^LYVE-1^+^ HEVs.^41^ Meanwhile, LTβR is also detected on ECs, and the absence of its ligand, LTα1β2, attenuates lymphatic vessel formation in TLSs.^319^ LIGHT also binds to LTβR on ECs, causing them to convert to HEVs. LIGHT-VTP normalizes tumor vasculature and triggers lymphoid aggregate.^36^ The AAV-LIGHT therapy modulates the vascular phenotype, eliciting the formation of HEVs and TLSs, which prolong the survival of mice with gliomas resistant to anti-PD-1 treatment.^320^ Furthermore, HEV neogenesis also relies on TNFR signaling. Blockade of TNFR by TNFRII.FC reduces the expression of PNAd and MAdCAM-1 in ECs, leading to HEV destruction. Notably, Fleig et al. found that conditional deletion of Rbpj, a key mediator of Notch signaling, led to a significant downregulation of the Notch pathway in vascular endothelial cells, inducing arterial endothelial cells to transform into HEV. In addition, they also observed the spontaneous formation of mature TLSs in the kidney, liver, and lung. This suggests that HEVs originate from vascular endothelial cells rather than lymphatic endothelial cells and are independent of SLOs.^321^

Several studies have shown a positive association between HEVs and prognosis in various tumors, such as colon carcinoma,^316^ oral cancer,^322^ carcinoma of the lungs,^323^ and breast cancer.^324^ For example, in a study of 203 samples with colon carcinoma, HEVs express sulfated L-selectin ligand, and patients with higher HEV/TLS ratios have longer overall and disease-free survival.^316^ Increased HEV frequency and maturation were associated with T-cell infiltration in tumors. They significantly improved ICB efficacy, especially survival after anti-PD-1/anti-CTLA-4 combination therapy.^325^ In addition, it has been reported that ICB treatment also promotes HEV formation, especially combined with antiangiogenic treatment, which may be due to the infiltration of immune cells.^326^ Allen E. et al. reported that anti-VEGF/VEGFR2 treatment induces HEVs and increases PD-L1^+^ T cells in MC38-bearing or glioma mice. Therefore, an anti-angiogenic combination with anti-PD-L1 induces a plump morphology of ECs and typical characteristics of MECA79^+^ HEVs.^282^ In human breast cancer, DC-LAMP^+^ DCs are found close to HEVs,^327^ and depletion of DCs attenuates HEV development,^328^ suggesting that DCs may also regulate HEVs through LTα1β2. Moreover, CD8^+^ T cells are correlated with the HEVs in TLSs, as evidenced by the absence of HEVs in anti-CD8-treated mice.^13^ T cells express TNF-α and LTα3 in FOXP3^+^ Treg deficiency mice,^329^ indicating that increased activated CD8^+^ T cells promote intertumoral HEV formation in a TNFR and LTβR-dependent manner.^136^

#### HEVs promote lymphocyte transport

Naive lymphocytes migrate into lymphoid organs through HEVs and undergo four key processes: rolling, adhesion to HEVs, intraluminal crawling, and transendothelial migration.^242,330,331^ HEVs express PNAd, a highly sulfated glycoprotein expressed on HEV-ECs.^332,333^ This protein translational modification enhances the combination of L-selectin^+^ lymphocytes with HEV-ECs. Chemokines induce the expression of integrin on lymphocytes, stopping lymphocyte rolling when they bind with an adhesion molecule (ICAM-1, ICAM-2) on ECs. Subsequently, these cells cross the HEVs and are recruited to a specified area formed by FRCs.^334^ More importantly, HEVs express CCL19 and CCL21, which bind to CCR7 on the surface of T cells and mediate T cell entry into lymphoid tissues.^335^ In addition, they also express CXCL13, which activates α4 integrins to induce B cell adhesion to MAdCAM-1^+^ HEV.^336^ Further study into HEVs revealed differences between MECA79^+^ HEVs within TLS^+^ and TLS^−^ regions in tumors. The HEVs upregulated TSPAN7 and MEOX2 gene expression in TLSs, which increased T cell and B cell infiltration and survival rates.^334^

### Differentiation of B cells and GC maturation

Human B cells are derived from hematopoietic stem cells and differentiate from pro-B cells to pre-B cells in the bone marrow, eventually becoming immature B cells. The early development of B cells involves the functional rearrangement of immunoglobulin gene fragments. Initially, the Ig heavy chain is constructed on the surface of pro-B cells, and the recombination-activating gene facilitates the rearrangement of V (VH), D (DH), and J (JH) gene segments in the variable region. Subsequently, the λ or κ light chain is rearranged and pairs with the heavy chain to form the B cell receptor (BCR) complex. The complete IgM molecules are expressed on the cell surface, indicating the cells have developed into immature B cells. Upon encountering specific antigens, B cells undergo affinity maturation and isotype switching, developing into memory B cells or antibody-producing PCs. This process occurs in the germinal centers of peripheral lymphoid tissue.^337^

**Fig. 5:**
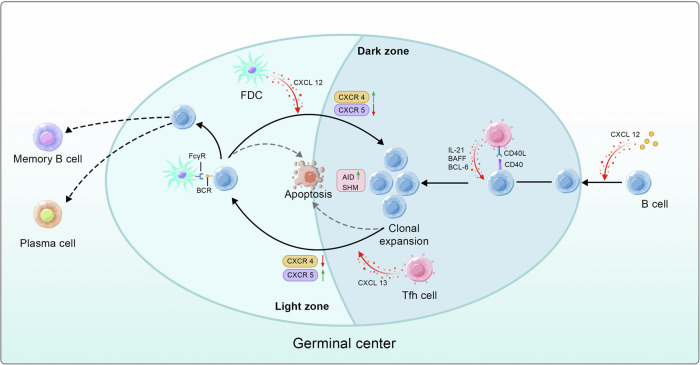
Differentiation of B cells, and GCs response. The diagram illustrates that GC is divided into two distinct compartments, with B cells entering the DZ under the influence of the CXCL12-CXCR4 axis, where GC-B cells proliferate and SHM occurs. Subsequently, B cells migrate along the CXCL13 gradient and enter the LZ. B cells capture antigens presented on FDCs for selection and immunoglobulin isotype switching. This process is also regulated by Tfh cells. B cells further proliferate, and SHM occurs through multiple migrations between the DZ and LZ. Eventually, they exit the GCs as memory B cells or high-affinity antibody-secreting plasma cells

Nascent GCs in the follicles were first detected a few days after immunization and were fully established within 1 week.^338^ Mature GC exhibits a distinct structure, comprising the peripheral light zone (LZ) and the inner dark zone (DZ). GC mediates humoral immunity by somatic hypermutation (SHM) and isotype switching, enabling B cells to generate high-affinity antibodies. Stromal cells and immune cells produce the chemokines CXCL12 and CXCL13, mediating the migration and localization of B cells.^339,340^ (Fig. 5) Pre-B cells are recruited into the DZ by HEVs after initially developing in the bone marrow.^284^ DZ-B cells undergo clonal expansion in the immunoglobulin variable zone.^341^ Negative selection eliminates self-reactive GC-B cells through SHM under the stimulation of antigen.^342,343^ Those processes increase their affinity for antigen and favor GC-B cell differentiation into proliferative PCs, promoting antibody production.^344^ After SHM and proliferation, B cells shift chemokine receptor expression: down-regulating CXCR4 and upregulating CXCR5, which directs them towards the LZ in response to CXCL13.^345^ The maturation of LZ-B cells increases antigenic affinity through positive selection, and lower-affinity B cells are deleted.^346,347^ Subsequently, CXCL12 promotes high-affinity B cells to reenter the DZ for clonal proliferation.^258,348^ During migration from the LZ to the DZ, partial B cells are eliminated.^349,350^ Mayer et al. reported that B cells express c-Myc, a positive selection marker, which helps them to evade death during migration.^351^ Additionally, BCR signaling triggers positive selection and enhances B cell survival in GC.^352^ However, this process requires antigen presentation by FDCs and CD40 signaling from Tfh cells, as BCR signaling alone causes B cell death.^353–355^ Tfh cells are crucial in driving B cells toward PC differentiation by costimulating factors. In vitro studies demonstrate that anti-CD40 induces the nuclear translocation of the NF-κB subunit p65/RelA in B cells.^356^ Interestingly, blockade of CD40L does not significantly affect Blimp1 expression in GC-B cells,^357^ suggesting that B cell differentiation may be regulated by multiple mechanisms. Besides, GC-B cells are characterized by the expression of B cell lymphoma 6 (BCL6) and Ki67.^58,358^ The absence of BCL6 leads to the failure of GC formation and high-affinity antibody production.^285,359^ FRCs are located at the boundary of the T-cell zone and B-cell zone, producing BAFF to maintain B-cell survival and GC structure.^360^ AID, a mutant enzyme, is critical to B cell maturation in DZ-B cells.^361,362^ Meanwhile, SEMA4A appears in TLS^+^GC^+^ HNSCC, suggesting that SEMA4A is correlated with B cell differentiation and TLS formation.^16^ Interestingly, the receptor for SEMA4A is TIM2, which is expressed on the surface of activated T cells.^363^ This seems to suggest that GC-B cells enhance T-cell function in TLSs.

The GC-B cells in TLSs overlap with those from healthy tonsils,^16^ indicating GCs are sites of B-cell expansion, maturation, and isotype switching in the TLSs. And GC formation is one of the indicators of TLS maturity.^364,365^ Analytical studies of B cell profiles within TLSs provide direct evidence of intact B cell responses, particularly in tumors. It has been shown that B cell density is higher in tumors with TLSs, and B cell density and function are also close to TLS presence.^59^ In breast cancer, Wang et al. analyzed B cell differentiation trajectories in the TME. They found that B cells transitioned from the transitional state of naive B cells and CXCR4^+^ B cells into follicular B cells and PCs, which are key components of GC-B cells. Notably, IgHG1 levels increased in the later stages of the pseudo-time trajectory, indicating that B cells underwent immunoglobulin conversion to become plasma cells.^366^ B cell immunity can arise from reactivating memory B cells or stimulating naive B cells. Naïve B cells and memory B cells are transported to the TME via HEV. A study indicated that naive B cells are primarily detected within TLSs, suggesting TLSs as the source of intratumoral B cells.^170^ However, another study noted memory B cell concentrations in the GC for in situ tumor activation, contrasting with scarce naive B cells.^367^ We think that elevated memory B cell levels may reflect the in situ maturation of naive B cells. This perspective is also supported in NSCLC.^59^ Spatial transcriptomics revealed that genes involved in all B cell maturation processes, from naive B cells to PCs, are present in TLS^+^ tumors. Notably, these gene signatures are preferentially expressed in TLSs.^367^ In TLS^high^ tumors, SHM levels, including BCR IgVL and IgVH, were significantly elevated compared to TLS^low^ tumors. Furthermore, TLS^+^ tumors exhibited a high density of Ig-producing PCs and Ig antibodies, especially IgG. In fact, the IgH clonotype was found both near and distant from the TLSs within tumor nests. This suggests that B cells undergo hypermutation in TLSs and that MZB1^+^ PCs migrate into the tumor nest along with CXCL12^+^ fibroblast networks after maturation.^367^ In multiple sclerosis, IgG-VH transcripts exhibit a high frequency of somatic hypermutation (SHM). SHM is a necessary stage for B cell affinity maturation, indicating the occurrence of GC responses within meningeal TLSs.^53^

## The role of TLS in the disease

TLSs represent crucial immune tissues capable of eliciting antigen-specific immune responses at sites of chronic inflammation. Their roles across different disease contexts and their implications for patient outcomes are diverse. In the realm of autoimmune diseases and age-related chronic inflammatory conditions, which are typically characterized by the immune system’s attack on self-tissues, the emergence and maturation of TLSs are often associated with poorer prognoses. Conversely, in scenarios necessitating an enhanced immune response, such as in cancer and infectious diseases, the presence and maturation of TLSs generally signify more favorable outcomes.^368–370^ Additionally, ageing serves as a critical influencing factor in the formation of TLSs induced by diseases. Various studies have demonstrated an age-dependent probability of TLSs formation across multiple diseases, including bladder cancer and acute kidney injury. This trend underscores the significant role of ageing in modulating the immune landscape and disease pathology through the promotion of TLS development^371–374^ (Table 2).

**Table 2:** TLSs in diseases: detection, markers, components, location, prognostic value, and therapeutic effects

Disease		Detection	Markers	Components	Location	Prognostic Value	Therapeutic Effects	Ref
Tumor	HNSCC	scRNA-seq	NA	TIL-B cell with GCs TLSs with GCs	NA	Positive	NA	^16^
	HNSCC	IHC	LAMP3 CCL2 CCL3 CCL4 CCL5 CCL18 CCL19 CCL21 CXCL9 CXCL10 CXCL11 CXCK13 CXCR4	Memory B cells Immature and activated B cells memory and activated CD4 ^+^ T CD8 ^+^ T cells activated dendritic cells type I T helper cells MDSC	Intra-tumor	Positive	NA	^380^
	HNSCC	IF FC	CD3 CD8 FoxP3 CD20 cytokeratin	Cytotoxic CD4^+^ T helper cells Regulatory T cells (Tregs) B lymphocytes	NA	Positive	PD-L1 positive	^484^
	HNSCC	scRNA-Seq	CXCR3 CCR7 CCR6 CXCR5 CCR1	NA	-	Positive	ICB positive	^368^
	TSCC	IHC	IL10 CD19 Foxp3 CD4	Bregs Tregs		Negative	NA	^563^
	Ovarian Cancer	IF IHC RtPCR ELISA Western Blot	CD20 CD4 SCD1 CD8α CXCL13 CCL21 VEGFC	CD4 T cell CD20 B cell	NA	Positive	PD-1 positive	^376^
	HGSOC	MCPcounter	NA	TLS	NA	Positive	NA	^401^
	HGSOC	IHC RNA ISH double staining	CXCL13	CD4 T cell	NA	Positive	NA	^254^
	ESCC	H&E	CD20 Ki67 CD21 CD4 LAMP3 CD8	GC	NA	Positive	NA	^152^
	ESCC	mIHC H&E	CD21 CD23 PD-1 CD1c CD4 CD8 CD19 CD21 CD138	TLSs	peri-tumor	Positive	PD-1 positive	^383^
	LUAD	H&E	NA	TLSs	NA	Positive	NA	^369^
	LUAD	IHC	CD8 Foxp3 CD20 CD4 DC-Lamp CCL19 CCL21 CCR7	DC TLS	NA	Positive	NA	^399^
	LSCC	H&E IHC IF	CD21 CD23 CXCL13	GC TLS	NA	Positive	Neoadjuvant treatment negative	^5^
	NSCLC	H&E IHC	CD3 CD20 CD21	FDC T cell B cell	NA	NA	Neoadjuvant treatment positive	^397^
	NSCLC	scRNA-seq	CD79A CD83 CD69 SELL BANK1 HMGA1 AICDA RGS13	GC Follicular B cells Plasma cells	NA	Positive	ICB Positive	^397^
	NSCLC	FC ELISA	CD14 CD38 CD138 CD27	GC Memory B cell	NA	Positive	Neoadjuvant treatment positive	^59^
	nmCRC	IHC H&E	CD20 CD3 CD21 CXCL13 HEV	B cell T cell FDCs GC HEV	peri-tumor	Positive	NA	^384^
	nmCRC	mIF	CD21 CD23 CXCL13	GC FDC	NA	Positive	NA	^404^
	CRC	IHC	CD3 CD8 GKA-DR CD31 MECA-79	CD3 T cell CD8 T cell M1 macrophages Vessels HEV	Peri-tumor HEV/TLS	Positive	NA	^316^
	HCC	Pathological examination Gene expression profiling	CCL2 CCL4 CCL5 CCL8 CCL18 CCL19 CCL21 CXCL9 CXCL10 CXCL11 CXCL13	TLS	Intra-tumor	Positive	NA	^19^
	HCC	H&E	NA	TLSs	Peri-tumor	Negative	NA	^385^
	HCC	FFPE	NA	TLSs	Intra-tumor	Positive	NA	^406^
	CCA	H&E mIHC	CD20 CD21	TLSs	Intra-tumor	Positive	NA	^407^
	CCA	mIHC H&E	CD20 CD3 MECA-79 CD21 CD23 TCL1A PAX5 TNFRSF13C CD79A	TLSs	Intra-tumor	Positive	NA	^386^
	CCA	mIHC H&E	CD20 CD3 MECA-79 CD21 CD23 TCL1A PAX5 TNFRSF13C CD79A	TLSs	Peri-tumor	Negative	NA	^386^
	iCCA	mIHC H&E	CD3 CD8 CD20 CD68 CK19 PDL1 CD4 Foxp3 Bcl6	TLSs	Intra-tumor	Positive	NA	^382^
	iCCA	mIHC H&E	CD3 CD8 CD20 CD68 CK19 PDL1 CD4 Foxp3 Bcl6	TLSs	Peri-tumor	Negative	NA	^382^
	iCCA	mIF IHC	MECA-79 CD8 CD20	TLSs	NA	Positive	NA	^410^
	cHCC-CCA	Spatial transcriptome sequencing RNA-seq IMC IHC mIF	CK19 HepPar-1 GPC3	TLSs	Intra-tumor	Positive	NA	^564^
	ccRCC	IHC	CD3 Bcl6 CD8 CD10 Foxp3	TLSs	NA	Negative	Negative	^377^
	ccRCC	IHC H&E	CD3 CD20 CD21 CD23	TLSs	Tumor-proximal	Positive	NA	^412^
	ccRCC	IHC H&E	CD3 CD20 CD21 CD23	TLSs	Tumor-distal	Negative	NA	^412^
	ccRCC	IHC	CD3 CD8a CD20 CD4 CD68 CD31 Pan-Keratin Ecad αSMA VIM	T cells B cells Macrophages tumor cells epithelial cells endothelial cells fibroblasts	NA	Positive	NA	^135^
	BCa	Pathological examination	CXCL13	NA	NA	ICB positive	NA	^256^
	BCa	TCGA	NA	TLSs	NA	Negative	ICB negative	^413^
	MIBC	TCGA	NA	TLSs	NA	Positive	ICB positive	^414^
	UCB	IHC	CD20 CD3 Bcl-6 CD21	TLSs GC	NA	Positive	NA	^288^
	EVaGC	mIHC	CD20 CD21 CD4 CD8 Foxp3	TLSs	Intra-tumor	Positive	NA	^417^
	GC	mIHC	MECA-79 CD21	FDC HEV	NA	Positive	NA	^418^
	Bladder Cancer	scRNA-seq	NA	TLSs	NA	Positive	NA	^366^
	Bladder Cancer	TCGA	NA	TLSs	NA	Positive	NA	^420^
	Bladder Cancer	IHC	CD4 CD8 CD163 Foxp3	TLSs	Peri-tumor	Negative	NA	^387^
	TNBC	H&E IHC	CD8 CD45 CD20	Cytotoxic T cells Memory T cells B cells	NA	Positive	NA	^421^
	Breast Cancers	IHC	CD3 CD20 CD23	T cell B cell FDC	NA	Positive	NA	^422^
	EC	TCGA	NA	TLSs	NA	Positive	NA	^424^
	EC	IHC	CD8 CD20 CD4 CD38 CD23	TLSs	Intra-tumor	Positive	NA	^425^
	Glioma	TCGA	NA	TLSs	NA	Positive	NA	^381^
	Melanoma	mIHC	CD8 CD20 KIT67 CD4	TLSs	NA	Positive	NA	^388^
	Melanoma	scRNA-Seq IHC	CD20 CD40	CD20CD22ADAM28 B cell	NA	NA	ICB positive	^565^
	ePM	IHC scRNA-Seq	NA	TLSs	NA	Positive	NA	^431^
	EMPM	FFPE	NA	TLSs	NA	NA	Positive	^432^
	PDAC	scRNA-seq mIHC FC	CD3 CD21 CD103 CD8 CD20 PNAd BCL6 Ki67	CD8 T cell IgG1 class-switched memory B cell Memory CD4 T cell Naive B cell NK cell GC	NA	Positive	NA	^77^
	PDAC	scRNA-Seq mIF	NA	TLSs	NA	Positive	Chemotherapy positive	^277^
	PDAC	H&E mIHC	CD4 CD8 CD20 CD11c CD15 CD68 Foxp3	CD4T cells CD8T cells B cells DCs Granulocytes Treg Macrophages	NA	Positive	NA	^427^
	PDAC	IHC	CD4 CD8 CD20 CD45 CD68 Foxp3	TLSs	NA	Positive	NA	^428^
	cSCC	IHC H&E	CD3 CD8 CD 21 MECA-79	B cells T cells HEV FDC	NA	Positive	NA	^379^
	MCC	IHC RNA-seq	CD3 CD8 CD20 CD21	T cells B cells	NA	Positive	NA	^430^
Autoimmune disease	SLE LN	IHC	NA	TLSs	NA	NA	NA	^196^
	SLE LN	IHC	CD20 CD3 CD21	B cells T cells FDC	Kidney	Negative	NA	^439^
	SS	FC RNA-seq	NKp30 CD337	NCR3	Salivary glands	NA	NA	^182^
	SS	ELISA RT-PCR	CX3CL1	CX3CL1	Salivary glands	NA	NA	^267^
	RA	IHC IF	CD3 CD20 CD138 CD68	MC B cells	Synovial tissue	Negative	NA	^436^
	MS	FC RNA-seq	NA	TLSs	Meninges	NA	NA	^443^
	MS	IHC IF PCR	CD4 CD8 CD79a IBA1 CD68	TLSs	NA	NA	NA	^445^
	Uveitis	IHC	CD3 CD20 CD21	B cells T cells FDC	Retina	NA	NA	^291^
	Uveitis	scRNA-seq	NA	TLSs	Retina	NA	NA	^448^
	GCA	IHC IF	CD3 CD20 CD21 CD68 CD138	T cells B cells FDC Macrophages Plasma cells	Aortic	NA	NA	^450^
	GCA	IHC PCR IF	CD3 CD20 CD21	T cells B cells FDC	Temporal artery	NA	NA	^51^
	Pemphigus	IHC	CD3 CD20 CD138	T cells B cells Plasma cells	NA	NA	NA	^292^
	Pemphigus	IHC RNA-seq	CD4 CXCL13	Tregs Memory T cells	NA	NA	NA	^137^
Transplant	Heart transplant	IHC	CD3 CD45 MECA -79	T cells B cells PNAd	Heart	Negative	NA	^455^
	Heart transplant	IHC	CD20 CD27	TLSs	Epicardial coronary arteries	Negative	NA	^62^
	Kidney transplant	IHC H&E	CD20	TLSs	Kidney	NA	NA	^457^
	Kidney transplant	IHC	CD20	TLSs	Kidney	Positive	NA	^456^
	Lung transplant	IHC H&E	NA	TLSs	Lung	Positive	NA	^458^
Infection	Colitis	IF	B220 MHCII CD11c- CD3 CD11c MHCII^+^ CD35 CD31 MAdCAM-1	B cells T cells DC FDC Vessel HEV	Colon	No effect	NA	^459^
	Crohn’s disease (CD)	IF	CD20 CD3 IL-33R CD138 PNAd	B cells T cells innate lymphoid cells plasma cells	Lymphatic vessels	NA	NA	^370^
	CD	IHC	CD20 CD3 CD31 gp38 PNAd CD21 CD19	B cells T cells Vessel Lymphatic vessels HEV FDC	Transmural lesions	Negative	NA	^460^
	COPD	IHC	CD19	TLSs	SIgA- airway	Negative	NA	^179^
	COPD	IHC	CD20 CD21	B cells FDC	Distal lung tissue (peribronchiolar and parenchymal)	No relation	NA	^461^
	COPD	IHC	IL-18 CD68	GC	NA	Negative	NA	^462^
	Chronic rhinosinusitis with nasal polyps (CRSwNP)	IHC/IF	CD3 CD20 CD21 Ki-67 PNAd D2-40 CD11c CD138 CD68 CD56	T cells B cells FDC Proliferating B cells HEV Lymphatic vessels DC Plasma cells Macrophages NK cells	NA	Negative	NA	^464^
	Tuberculosis (TB)	IHC	B22 CD3	B cells T cells	NA	Positive	NA	^466^
	Influenza virus infection	IHC PNA staining	B220 CD11c CD4 CD8 M2 CD138 GL-7	B cells DC T cells FDC Plasma cells GC	Bronchi Lung interstitium	Positive	NA	^177^
	Influenza virus infection	HE IHC/IF	B220 CD3 CD11c GL7	B cells T cells DC GC	Bronchi	Positive	NA	^197^
	Staphylococcus aureus lung infection	IHC	CD20 CD3 PNAd CD21 PCNA	B cells T cells HEV FDC GC	Bronchi	No relation	NA	^467^
	Viral Myocarditis (VMC)	HE IHC/IF	CD3 B220 CD21 Ki67 PNAd	T cells B cells FDC HEV	NA	Negative	NA	^469^

### Tumor

TLSs are associated with tumor progression, immune responses, and patient prognosis. They might contribute to understanding cancer and developing new treatment strategies.^375–377^

TLSs are intricately linked to the prognosis of tumors. The location (intra-tumor or peri-tumor) and the maturation of TLSs can greatly influence the prognosis of cancer.^378–381^ In the majority of investigations, when TLSs are located intra-tumorally, patients with cancer often exhibit an improved prognosis.^380,382^ However, when TLSs are located peri-tumor, the prognosis of cancer patients shows strong heterogeneity. For instance, in cancers such as esophageal squamous cell carcinoma and colorectal cancer, patients with peri-tumor TLSs exhibited better outcomes,^383,384^ but in cancers such as hepatocellular carcinoma, cholangio carcinoma, and breast cancer, the outcomes were worse.^385–387^ Additionally, cancer patients with mature TLSs often exhibit a favorable prognosis.^277,367^

TLSs play a crucial role in enhancing adaptive immune responses. B cells in TLSs can produce specific antibodies and promote phagocytosis, complement-mediated cytotoxicity, or antibody-dependent cellular cytotoxicity, which may contribute to the adaptive immune defense against tumors.^59,388^ Hans A. Schlößer et al. demonstrated that in cancer, specific T-cell responses were induced by the accumulation of CD86^+^ antigen-presenting B cells within GCs in TLSs. TLSs may facilitate adaptive immunity in cancer by providing an organized space that promotes interactions between immunity.^389^ Willard-Gallo et al. further demonstrated the interactions of immune cells within TLSs in the breast cancer immune microenvironment.^390^

In this section, we delineate the associations between TLSs and tumor prognosis, highlighting their heterogeneity across various cancer types.

#### Head and neck cancer

Head and neck cancer is a group of malignancies originating from the oral cavity, nasal cavity, pharynx, larynx, and other structures in the neck.^391^ Head and neck squamous cell carcinoma (HNSCC), the most predominant subtype among head and neck tumors, has shown a better prognosis and a greater response to immunotherapy when associated with TLSs. In patients with HPV-positive (HPV^+^) or HPV-negative (HPV^−^) HNSCC, an increased presence of TLSs has been observed.^16^ Regardless of HPV status, the presence of TLSs with GCs correlates with favorable prognoses, indicating a potential role in immune-mediated tumor control. Furthermore, the enrichment of TLSs in HPV^−^ HNSCC patients is associated with extended overall survival and heightened responsiveness to PD-1 inhibitory treatment.^392^ These findings underscore TLSs’ significance in enhancing antitumor immunity and improving patient outcomes in the context of HNSCC, particularly in the HPV^−^ subgroup.^368^ Additionally, a specific subtype of TLSs, known as follicular-like tertiary lymphoid structures (FL-TLSs), serves as an independent prognostic marker in laryngeal carcinoma. Patients with FL-TLSs exhibit increased immune cell infiltration and demonstrate markedly improved responses to immunotherapeutic approaches, further highlighting the potential of TLSs as a biomarker for predicting treatment outcomes and guiding therapeutic strategies in HNSCC.^393^ Overall, these studies emphasize the crucial role of TLSs in modulating the immune landscape within HNSCC tumors and suggest their utility as prognostic indicators and therapeutic targets for improving patient outcomes.

#### Esophageal cancer

Originating from the mucosal lining of the esophagus, it falls under the category of cancers of the digestive system.^394^ Furthermore, current research indicates that the presence of TLSs both within the tumor itself and in the surrounding tissue is associated with a more favorable prognosis. Recent studies have consistently demonstrated that the presence of TLSs, particularly mature TLSs, within ESCC is closely associated with improved prognostic outcomes for patients. Mature TLSs are characterized by a rich composition of proliferating B cells, plasma cells, CD4^+^ helper T cells, B memory cells, and Th17 cells, and are correlated with an increased infiltration rate of CD8^+^ T cells.^152^ This heightened infiltration is intimately linked to enhanced survival rates. Furthermore, cases featuring mature TLSs significantly outperform others in terms of recurrence-free survival (RFS) and overall survival (OS).^395,396^ Additionally, a high density of peri-tumoral TLSs is associated with earlier tumor stages, the absence of lymphovascular invasion, improved serum nutritional parameters, and extended survival periods. Remarkably, the density of TLSs can also serve as a predictive marker for the response to PD-1 inhibitor therapy and survival outcomes.^383^ The convergence of findings across multiple studies underscores the critical clinical relevance of considering the tumor microenvironment, especially the assessment of TLSs, in the treatment and prognostic evaluation of ESCC. This emphasizes the pivotal role of TLSs in cancer progression and treatment response.

#### Non-small cell lung cancer

Lung cancer, one of the most prevalent cancers worldwide, originates from the cells within the lungs and stands as a leading cause of cancer-related mortality globally. It is broadly categorized into two main types based on its origin and cellular characteristics: non-small cell lung cancer (NSCLC) and small cell lung cancer (SCLC). NSCLC accounts for approximately 85% of all lung cancer cases and is characterized by a relatively slower progression. It primarily encompasses three subtypes: adenocarcinoma, squamous cell carcinoma, and large cell carcinoma. Multiple studies underscore the pivotal role of TLSs in enhancing prognoses and therapeutic outcomes in NSCLC patients. It has been demonstrated that in NSCLC, the maturity and abundance of TLSs significantly influence the efficacy of neoadjuvant chemotherapy, with patients exhibiting higher TLS maturity and quantity experiencing improved DFS.^397^ This suggests that fostering the maturation of TLSs could be a novel approach to boosting the effectiveness of neoadjuvant chemotherapy.

Similarly, analysis of data from 891 NSCLC patients treated with atezolizumab revealed increased activity of B cells and plasma cells, indicating their crucial role in extending overall survival, regardless of CD8 T cell characteristics. This was further confirmed through scRNA-seq, highlighting the significant impact of the specific organization of B cells and plasma cells within TLSs on treatment outcomes.^369^ In lung adenocarcinoma, an automated workflow for quantifying TLS density in whole-slide images established TLS density as an independent prognostic marker for patients with resectable disease.^369^ Furthermore, patients with mature TLSs exhibited significantly lower rates of lymph node metastasis, earlier cancer staging, and higher numbers of CD8^+^ and FOXP3^+^ cells in TILs, with mature TLSs being independently associated with better DFS and OS. In lung squamous carcinoma (LSCC), specific niches consisting of CXCL13^+^ perivascular cells and CXCL12^+^LTB^+^ and PD-L1^+^ epithelial cells were identified as facilitators for TLS formation, with TLS density serving as the strongest independent prognostic marker.^398,399^ However, the prognostic value of TLS density was compromised in patients receiving neoadjuvant chemotherapy, attributed to impaired GC formation.^5^ Additionally, preoperative corticosteroid treatment, regardless of chemotherapy, was found to reduce TLS density and GC formation, highlighting the complex interplay between treatment modalities and TLS dynamics in influencing cancer outcomes.^61^

#### Ovarian cancer

Ovarian cancer, a malignant tumor originating from ovarian cells, can be classified into three primary types based on the cell of origin: epithelial ovarian cancer, stromal tumors, and germ cell tumors. Of these, epithelial ovarian cancer is the most prevalent, representing approximately 90% of all ovarian cancer cases. Furthermore, epithelial ovarian cancer can be subdivided into several histological subtypes, with high-grade serous ovarian cancer being the most common.^400^ Research on TLSs in ovarian cancer indicates their association with favorable prognoses. It has been discovered that CDK4/6 inhibitors (CDK4/6i) can promote the formation of TLSs and enhance the efficacy of anti-PD-1 antibody immunotherapy, potentially through the regulation of SCD1 and its modulatory molecules ATF3 and CCL4.^376^ Additionally, another study investigated the distribution and gene expression of TLSs in high-grade serous ovarian cancer (HGSOC) samples, finding that the expression of the CXCL13 gene is not only closely related to the presence of TLSs but also associated with the infiltration of T and B cells, suggesting that CXCL13 is a marker of favorable prognosis in HGSOC patients. These studies reveal that the coexistence of CD8 T cells and B cells in the tumor microenvironment (TME) significantly improves the prognosis of HGSOC, which is associated with the presence of TLSs.^254^ The expression of CXCL13 transitions to CD21^+^ follicular DCs as TLS matures, initially emanating from CD4 T cells. Furthermore, patients with HGSOC showing high expression of TLS exhibit significantly better progression-free survival (PFS), linked to the maturation of B cells and the activation and proliferation of tumor-specific T cells.^401^ These findings underscore the critical role of TLSs in the treatment and prognostic assessment of ovarian cancer, offering potential targets for future immunotherapy strategies.

#### Colorectal cancer

Colorectal Cancer (CRC), originating from the glandular tissues of the colon’s mucosa, ranks third globally in both incidence and mortality rates among cancers. Preventative measures and early screening are crucial in mitigating its epidemiological impact.^402^ Several studies in CRC and colorectal liver metastases (CRCLM) have underscored the role of TLSs and their association with patient prognosis. One study observed significantly higher densities of Tregs, M2 macrophages, and Tfh cells within intra-tumoral TLSs compared to those at the tumor periphery. Intra-tumoral TLSs were positively associated with RFS and OS, whereas peritumoral TLSs correlated negatively with RFS and OS.^403^ Another study identified that a high density of peri-tumoral TLSs, combined with a low tumor stroma percentage, serve as an independent and favorable prognostic factor in non-metastatic colorectal cancer (nmCRC) patients. This finding emphasizes the significance of tumor microenvironment characteristics in influencing the prognosis of nmCRC patients.^384^ Furthermore, the presence of mature TLSs is linked to a favorable prognosis in CRC patients, particularly those with nmCRC.^404^ Additional research highlighted that CRC tissues with high levels of HEVs within TLSs demonstrated enhanced recruitment capabilities for CD3 T cells, CD8 T cells, and macrophages. This was positively associated with improved OS, DFS, and lower TNM staging, suggesting that high HEV/TLSs could serve as potential biomarkers for CRC patients.^316^ In summary, the density and location of TLSs, along with the cellular composition within these structures, significantly impact the prognosis of CRC and CRCLM patients. This highlights the critical prognostic value of TLSs within the tumor microenvironment, underscoring their potential as therapeutic targets and biomarkers in CRC management.

#### Hepatocellular carcinoma

HCC represents the most prevalent form of liver cancer globally, commonly associated with chronic liver diseases and cirrhosis, constituting a significant oncological challenge.^405^ In the study of HCC patients undergoing surgery, an increased number of TLSs in non-tumor liver tissues was observed to correlate with a higher likelihood of late recurrence and a shorter overall survival period.^385^ Contrasting this, research into the presence of TLSs within tumors found that patients harboring these structures demonstrated a significantly reduced risk of early postoperative recurrence, suggesting that intra-tumoral TLSs may serve as indicators of effective anti-tumor immune responses.^19^ Furthermore, analysis revealed that HCC patients with high intra-tumoral TLS content exhibited significantly improved RFS and DFS and identified characteristic genes associated with TLS positivity. This provides potential predictive markers for prognosis.^406^ These findings collectively underscore the pivotal role of TLSs in hepatocellular carcinoma, revealing that the location of TLS presence (intra-tumoral versus peri-tumoral tissue) has a significant impact on patient outcomes. These discoveries not only enhance our understanding of the immune composition within the tumor microenvironment but also offer new avenues for future therapeutic strategies, especially in designing treatments aimed at activating or enhancing intra-tumoral immune responses. Moreover, the identification of genes associated with TLS positivity opens avenues for personalized medicine and precision treatment, aiding in the identification of HCC patients who may benefit from specific immunotherapies.

#### Cholangiocarcinoma

Cholangiocarcinoma (CCA) represents a relatively rare yet severe form of cancer originating from the bile duct cells. The bile ducts are a series of small tubes tasked with the transport of bile, produced by the liver, to the small intestine, facilitating the digestion process. Based on the location of the cancer’s onset, CCA can be categorized into two main types: intrahepatic and extrahepatic CCA, with the latter further subdivided into proximal and distal extrahepatic CCA. In the investigation of extrahepatic CCA, this study delineated and categorized TLSs into lymphoid aggregates, primary, and secondary follicle-like configurations. Notably, the presence of intra-tumoral secondary follicle-like TLSs was significantly associated with enhanced overall survival and improved recurrence-free survival rates.^407^ Key markers, including PAX5, TCL1A, TNFRSF13C, and CD79A, were identified as positive indicators for the presence of TLSs within CCA, suggesting that intra-tumoral TLSs portend a favorable prognostic outlook. In contrast, TLSs situated in the peri-tumoral region were linked to negative clinical outcomes, marked by an increased frequency of satellite lesions, lymph node metastasis, and a higher incidence of fatty liver disease.^386^ Extending the scope to intrahepatic CCA (iCCA), a multicenter analysis elucidated that an augmented presence of TLSs within the tumor is correlated with superior overall survival, whereas proliferation of TLSs in the peri-tumoral area is associated with diminished overall survival.^382^

The abundance of TLS within tumors is identified as an effective prognostic marker for favorable outcomes in iCCA, while the presence of peritumoral TLS significantly correlates with adverse outcomes. This dual functionality of spatially distinct TLS, also recognized in hepatocellular carcinoma and breast cancer, remains to be fully understood. Our findings reveal a marked increase in the frequency of CD4^+^BCL6^+^ T follicular helper (Tfh) cells within tumor-infiltrating TLS compared to those surrounding the tumor, underscoring the pivotal role of Tfh cells in the maturation and maintenance of germinal centers, thereby shaping the antitumor immune milieu.^408,409^ High levels of Tfh cells within tumor-associated TLS could be foundational to favorable prognostic outcomes. In contrast, a significant enrichment of CD4^+^FOXP3^+^ regulatory T (Treg) cells is observed within tumor-associated TLS compared to peritumoral ones, suggesting a potential for immune suppression. Moreover, as peritumoral TLS abundance increases, there’s a significant rise in the frequency of Treg cells within tumor-associated TLS, hinting at a potential linkage between peritumoral TLS and the tumor’s immunosuppressive environment.^134,385^ Given the previous insights that peritumoral TLS may reflect an inflammatory backdrop fostering tumor growth and serving as a niche for malignant cell migration, we postulate that the scarcity of Tfh cells, coupled with morphological observations, suggests peritumoral TLS might be in an immature and dysfunctional state, potentially compromising the antitumor immunity facilitated by tumor-infiltrating TLS. This investigation into the contrasting roles of TLS within and around tumors offers a deeper insight into the intricate dynamics of the tumor microenvironment, highlighting the sophisticated interplay between Tfh and Treg cells that could pave the way for novel therapeutic interventions targeting the immune landscape of tumors.^382^ The investigation further illuminated a notable elevation in T follicular helper cells and regulatory T cells within tumor-associated TLSs, revealing a sophisticated immune milieu that markedly influences patient prognosis. Additionally, the detection of HEVs in TLSs was significantly correlated with extended recurrence-free survival, thereby emphasizing the prognostic relevance of these immune structures within the tumor microenvironment.^410^ These collective insights accentuate the pivotal role of TLSs as prognostic indicators and potential therapeutic targets in CCA, underscoring the intricate nexus between tumor biology and immune system dynamics in shaping patient outcomes.

#### Renal cancer

Renal cancer ranks as the thirteenth most common cancer worldwide. Clear cell renal cell carcinoma (ccRCC) is the most common subtype of RCC, accounting for 70–80% of all RCC cases. Typically originating from the epithelial cells of the renal tubules, ccRCC holds a central position in the research, diagnosis, and treatment of renal cell carcinoma due to its high incidence and distinct pathological features.^411^ Research has unveiled that the presence of TLSs within the tumor microenvironment is linked to adverse post-surgical outcomes in ccRCC, as well as resistance to anti-angiogenic treatments upon disease recurrence. Comparative analyses between renal and bladder cancer samples have highlighted a persistent immaturity of TLSs in renal tumors, with mature follicle-like TLSs absent in the germinal centers of ccRCC. This discrepancy in TLS maturation may contribute to the observed variances in patient prognoses.^377^ Further investigations have elucidated the dual roles of TLSs in tumor progression, revealing that TLSs in ccRCC predominantly localize to distal tumor regions, exhibiting immature and immunosuppressive characteristics. Such localization is correlated with poorer PFS and OS.

Conversely, TLSs situated in proximal tumor regions are significantly associated with enhanced PFS and OS. Notably, the presence of TLSs bearing mature characteristics, particularly those containing CD23^+^ GCs, is closely linked to more favorable clinical outcomes in ccRCC patients.^412^ Analyses of TME in ccRCC and adjacent non-cancerous tissues have demonstrated significantly higher levels of CD8 T cells, CD163^+^ macrophages, regulatory T cells, endothelial cells, and fibroblasts in ccRCC tissues. The immune infiltration patterns within the TME exhibited clustered and dispersed configurations. Tissues harboring TLSs showcased interactions between CD8 T cells and B cells, including GZMB^+^ cells, indicative of antitumor properties and improved prognosis. On the contrary, the macrophage/T cell cluster phenotype was associated with poorer outcomes. For patients with dispersed immune structures, further classification based on immune core numbers (CN) identified dispersed CN-hot and CN-cold phenotypes, both of which displayed better prognoses than the macrophage/T cell cluster phenotype, albeit with distinct immune characteristics and potential treatment responses.^135^ Moreover, leveraging spatial transcriptomics, the critical role of TLSs within the context of RCC has been underscored, highlighting their significance in promoting antitumor immune responses. The study meticulously detailed the transformation process of B cells within TLSs into plasma cells, a maturation process culminating in the production of specific antibodies targeting tumor cells, thereby triggering apoptosis. Furthermore, these findings emphasize the indispensable role of intra-tumoral TLSs in amplifying anti-tumor immune mechanisms, offering profound insights for the development of future therapeutic strategies.^367^

#### Bladder cancer

Bladder cancer is a prevalent malignancy of the urinary system, posing a significant challenge to public health worldwide. Originating from the epithelial cells of the bladder, the most common type is urothelial carcinoma. In the domain of bladder cancer research, a recent discovery has unveiled that the expression of CXCL13 is significantly associated with both PFS and OS in patients undergoing immune checkpoint blockade (ICB) therapy. This association, however, does not extend to patients who have not received ICB therapy, underscoring the potential role of CXCL13 in predicting the efficacy of ICB treatments. Moreover, the expression of CXCL13 is closely linked to the presence of TLSs within tumors, further affirming its importance as a marker of TLSs in bladder cancer.^256^ Analysis also revealed that bladder cancer patients with high levels of TLSs exhibit better prognoses and higher immune cell infiltration compared to those with low TLSs. Nonetheless, patients characterized by low TLSs demonstrated superior responses to ICB therapy and conventional chemotherapy. These findings highlight the pivotal role of TLSs in determining therapeutic responses and prognoses in bladder cancer patients.^413^ Further investigations have shown that the presence of TLSs in patients with muscle-invasive bladder cancer (MIBC) correlates with higher survival rates and increased responsiveness to PD-1 blockade therapy.^414^ TLSs are significantly more prevalent in MIBC patients compared to those with non-muscle invasive bladder cancer (NMIBC). The presence of TLSs is closely associated with the formation of GCs and an increased density of TILs. Furthermore, analyses indicate that TLS presence is associated with enhanced DFS in both NMIBC and MIBC patients.^288^ Additionally, another study has shown that, in comparison to untreated tumors, the fifth category (low macrophage) of TLSs was significantly higher following pre-operative immunotherapy. These critical findings underscore the key role of TLSs in the treatment and prognosis of bladder cancer patients, offering invaluable insights for future research and therapeutic strategies.^415^

#### Gastric cancer

Gastric cancer ranks among the most prevalent cancer types globally and is characterized by a diversity of biological properties, histological classifications, and clinical manifestations. Adenocarcinoma emerges as the most common form among these variants.^416^ In a study focusing on patients with gastric cancer, it was discovered that the presence of mature TLSs within tumor tissues is significantly positively correlated with the overall survival rates of the patients. Further analyses revealed a close association between high levels of TLSs and several clinicopathological characteristics of the tumor, including tumor size, histological grade, and disease staging. These associative findings underscore the potential role of TLSs in the progression of gastric cancer and suggest their value as a biomarker for assessing patient prognosis.^417^ Subsequent research indicated that in patients with specific stages of gastric cancer, a high density of TLSs is closely linked to better survival outcomes, a finding that was confirmed through various statistical analysis methods. These results not only reinforce the importance of TLSs within the tumor immune microenvironment but also provide vital reference information for future clinical practice, highlighting the potential to improve prognosis in gastric cancer patients by enhancing TLS levels.^418^ These research findings enrich our understanding of the role of TLSs in gastric cancer, offering significant scientific evidence for the development of new treatment strategies and the improvement of patient management.

#### Breast cancer

Breast cancer, a malignant tumor originating from breast tissue, stands as the most prevalent cancer among women worldwide.^419^ A series of studies have elucidated the pivotal role of TLSs in breast cancer. It has been observed that there is a positive correlation between tumor-associated neutrophils and tumor-infiltrating B cells, with this correlation being more pronounced in patients with low TLS levels compared to those with high TLS levels. Furthermore, patients exhibiting mature TLSs demonstrated superior outcomes in both chemotherapy and immunotherapy.^366^ Analysis of the Cancer Genome Atlas breast cancer cohort revealed that the presence of TLSs is associated with improved DFS and OS. This association positively correlates with early tumor TNM staging, a high quantity of TILs, and the expression levels of Human Epidermal Growth Factor Receptor 2 and Ki-67, while exhibiting a negative correlation with the status of estrogen and progesterone receptors.^420^ This indicates that a high TLS signature is indicative of a more favorable TEM, leading to better survival outcomes. In triple-negative breast cancer, combining the assessment of TLSs with tumor budding (TB) showed a positive correlation with OS and RFS, suggesting that the TLS/TB model may surpass the classical tumor-lymph node metastasis staging system in predicting patient outcomes.^421^ Moreover, in the breast cancer tumor microenvironment, TLS presence is positively associated with high tumor grade, phenotype, necrosis, extensive in situ components, lympho-vascular invasion, and high levels of TILs, as well as with hormone receptor negativity, HER2 positivity, and c-kit expression. Notably, in HER2-positive breast cancer, the presence of TLSs significantly correlates with better DFS, independently of high TIL expression, indicating that the combined status of TLSs and TILs represents an independent favorable factor related to DFS.^422^

#### Uterine cancer

Uterine cancer, a malignancy originating from the cervix of the female uterus, represents a significant category of gynecological cancers that can be effectively managed through regular screening and timely intervention.^423^ Within the context of endometrial cancer patients, the presence of TLSs has been demonstrated to be a favorable prognostic marker, significantly impacting both recurrence timelines and cancer-related mortality rates. Specifically, patients with TLS exhibit a markedly reduced risk of recurrence within a 5-year period compared to those devoid of TLSs. Moreover, research indicates that mature TLSs serve as the optimal predictive markers when integrated with clinicopathological factors and molecular classifications, offering superior predictive value over densities of CD8 or CD20. Intriguingly, mature TLSs may express L1 Cell Adhesion Molecule (L1CAM), a feature not observed in immature TLSs, and the employment of L1CAM as a marker for mature TLSs achieves a high level of observer agreement.^424^ Further investigations have elucidated a negative correlation between the presence of TLSs and patient tumor progression, particularly noting a significant extension in progression-free survival when CD23^+^ GCs are contained within TLSs. Additionally, the quantity of CD20^+^ B cells within TLSs significantly surpasses that in patients without TLSs, with these B cell numbers positively correlating with other TLS components and associated with improved progression-free survival outcomes.^425^ Overall, the presence of TLSs and the infiltration of B cells within them are closely linked to more favorable survival outcomes in patients with endometrial cancer.

#### Pancreatic cancer

Pancreatic cancer, characterized by its highly lethal nature, originates from the cells within the pancreas and is frequently diagnosed at an advanced stage, presenting significant challenges for effective treatment.^426^ The presence of TLSs, whether nascent or mature, is associated with higher survival rates and favorable prognoses in pancreatic ductal adenocarcinoma (PDAC). Studies suggest that TLSs enhance adaptive immune responses within TEM. PDAC with TLSs is enriched with memory lymphocytes and IgG-switched B cells, which correlates with prolonged survival.^77^ Moreover, a subset of PDAC exhibits TLSs that support the proliferation and differentiation of B cells into plasma cells, and these mature TLSs also bolster T cell activity, being rich in tumor-reactive T cells. Chronic activation of tumor-reactive T cells may organize TLSs through the production of the B cell chemoattractant CXCL13.^277^ In PDAC patients undergoing chemoimmunotherapy, a gene signature reflective of mature TLSs in pretreatment biopsy samples correlates with extended survival. The presence of TLSs in patients receiving surgical treatment significantly correlates with improved PFS and OS, associated with increased infiltration levels of CD8^+^ T cells, CD4^+^ T cells, B cells, and activated immune cells.^427^ However, the prognostic value of TLSs is not significant in patients receiving neoadjuvant therapy. TLSs are associated with high levels of CD4^+^ TILs, CD8^+^ TILs, and CD45RO^+^ TILs but not with high levels of FOXP3^+^ TILs, correlating with longer pancreas-specific survival and favorable outcomes with adjuvant S-1 therapy.^428^ These findings highlight the potential of TLSs as prognostic markers and therapeutic targets in PDAC, revealing complex interactions between the tumor microenvironment and the immune system. They suggest that enhancing or mimicking the functions of TLSs could improve therapeutic outcomes for PDAC patients.

#### Skin cancer

Skin cancer is primarily categorized into three major types based on the cell of origin: basal cell carcinoma, squamous cell carcinoma, and malignant melanoma.^429^ Additionally, there exist rare yet highly aggressive forms of skin cancer, such as Merkel cell carcinoma. Current research on TLSs in skin cancer has predominantly focused on melanoma, squamous cell carcinoma of the skin, and Merkel cell carcinoma, reflecting a concentrated effort to understand the immunological landscape within these malignancies. In studies of cutaneous malignant melanoma, it has been found that the presence of TLSs in 47% of cases was associated with an increase in TILs, although the proliferation of lymphocytes within the TLSs did not correlate with TIL proliferation.^388^ The presence of TLSs was linked to a reduced risk of tumor recurrence and an improvement in OS, particularly in cases with a lower proportion of CD21^+^ B cells in TLSs.

Further research has shown that the presence of tumor-associated CD8^+^ T cells and CD20^+^ B cells correlate with better survival outcomes, a correlation that is independent of other clinical factors. Tumors with a high concentration of B cells also exhibited increased levels of TCF7, indicative of naive and/or memory T cells. In melanoma lacking TLSs, T cells displayed a dysfunctional molecular profile, underscoring the importance of TLSs in influencing the immune microenvironment and T cell phenotypes for an enhanced immune response.^378^ In Merkel Cell Carcinoma (MCC), analysis indicated that samples with TLSs had a significantly better prognosis compared to those without. Merkel cell polyomavirus (MCPyV) is a major causative agent of MCC. It contributes to the malignant transformation and oncogenesis of host cells through the integration of its viral genome into the host cell DNA and the action of viral proteins. Notably, the prognosis of MCPyV-positive samples was favorable regardless of TLS presence, but MCPyV-negative samples with TLSs had a prognosis similar to that of MCPyV-positive samples, highlighting the complex interplay between viral factors and immune structures in tumor environments.^430^ For Cutaneous Squamous Cell Carcinoma (cSCC), the presence of TLSs was significantly associated with better histopathological grades and higher levels of sun exposure. Additionally, the presence of intra-tumoral TLSs was linked to lower lympho-vascular invasion, suggesting that TLSs may play a protective role in the progression of cSCC.^379^ Collectively, these findings emphasize the critical role of TLSs in modulating the immune landscape across various skin cancers, potentially guiding more effective therapeutic strategies.

#### Other tumors

In studies of other tumor types, such as gliomas and epithelioid mesotheliomas, the presence of TLSs within the tumor microenvironment has been significantly correlated with a more favorable prognosis and a better response to therapy. These findings underscore the critical role of TLSs in the tumor immune microenvironment, suggesting that they may serve as key modulators of tumor biological behavior, influencing patient survival and response to treatment. Therefore, a deeper exploration of the functions and mechanisms of TLSs across different tumor types not only aids in our understanding of the complexities of tumor immune evasion but also lays the theoretical groundwork for developing novel immune therapeutic strategies based on TLSs. Furthermore, employing TLSs as biomarkers for clinical prognostication and personalized treatment decisions may pave a new pathway for enhancing the efficacy of cancer therapies.^381,431,432^

Recent studies have shown that not only the heterogeneity of TLS itself but also changes in cellular metabolic levels and the acidity of TME can affect TLS formation and function, thereby altering the immune niche. Hu et al. recently reported that T-cell glucose metabolism promotes TLS formation. In TLSs within advanced-stage gastric cancer, B cells produce LTα binding to TNFR2 on the surface of CXCL13^+^CD103^+^T cells, which activates tumor necrosis factor receptor-associated factor 2 (TRAF2), enhancing CXCL13 expression through the photoshatidylinositol-3 kinase (PI3K)/protein kinase B (AKT)/mTOR pathway. And mTOR is an essential glycolysis-regulating pathway.^299^ However, there appears to be a negative feedback regulatory function that prevents immune over-activation. Because an acidic environment inhibits T cell function. A study suggested that the pH of the T-cell area in the LN (6.3) was significantly lower than other sites (LN peripheral pH 7.1 and peripheral vessel pH 7.1).^433^ Due to aerobic glycolysis occurring in T cell activity leading to lactate accumulation.^434^ However, decreased pH inhibits T-cell lactic acid efflux and glycolysis, and suppresses cytokine release, such as IL-2 and IFN-γ, but not T-cell activation.^433^ Tumor cells are characterized by aerobic glycolysis, which suppresses T cell function. Additionally, T cells produce lactic acid, leading to a decrease in pH in TLS, which suppresses T cell-dependent immune function and even inhibits TLS development. More importantly, this probably explains the absence of mature TLS in some tumors. We speculate that, in the early stages of TLS development, tumors with high levels of glycolysis produce acid accumulating in the TME and decreasing in pH, which inhibits the LTo cell-like function of T cells and impairs the efficiency of TLS formation. Therefore, selectively manipulating the pH of tumor-associated TLSs may enhance T cell function, which is beneficial for immunotherapy. However, the effect of acidic TME on B cell function in TLS remains unknown, which also requires further study.

### Autoimmune diseases and immunoinflammatory

#### Rheumatoid arthritis

Rheumatoid arthritis (RA) is among the most prevalent autoimmune diseases, characterized pathologically by synovitis, marked by the infiltration of immune cells, proliferation of synovial fibroblasts, and neovascularization.^435^ RA can be categorized into three distinct pathological types: the lympho-myeloid type, featuring aggregates of B cells; the myeloid type, characterized by infiltration of macrophages in the synovial lining; and the pauci-immune fibroid, notable for its lack or minimal inflammatory cell infiltration.^55^ In the assessment of untreated RA cases, it was discovered that high synovial mast cell counts correlate with local and systemic inflammation, autoantibody positivity, and increased disease activity. These mast cells, situated on the periphery of lymphoid aggregates, were shown to play a significant role in promoting the activation and differentiation of naive B cells, as well as inducing the production of anti-citrullinated protein antibodies (ACPAs), primarily through contact-dependent interactions.^436^ Additionally, the presence of TLSs was associated with a more severe disease course in RA. In a separate evaluation, it was found that endothelial cells can produce NF-κB-inducing kinase, a key player in non-classical NF-κB signaling triggered by LTβ receptor signaling, which contributes to the formation of TLSs in RA.^437^ This highlights the complexity of cellular interactions and signaling pathways involved in the progression and severity of RA, underscoring the importance of TLSs and mast cells in the disease’s pathology.

#### Systemic lupus erythematosus and lupus nephritis

Systemic lupus erythematosus (SLE) is a chronic, multisystem, autoimmune disease characterized by clinical heterogeneity. Lupus nephritis is a form of glomerulonephritis that can occur in patients with SLE and is one of the most common and severe organ manifestations of the disease. Lupus nephritis is characterized by the deposition of immune complexes and inflammation of the renal tubulointerstitial area.^438^ A study found that in patients with lupus nephritis or SLE, the development of TLSs in the kidneys is a significant phenomenon. It was observed that under conditions of chronic inflammation, mesenchymal stem cells express various inflammatory markers and play a crucial role as lymphoid tissue organizers in accelerating the inflammatory process and initiating the formation of kidney-specific TLSs.^196^ Additionally, an analysis of SLE patients revealed that those with lupus nephritis exhibited different classes of disease severity based on the expression of CD20, CD3, and CD21 in the kidneys. The presence of CD20 and the formation of TLSs were associated with a longer disease course, a poorer response to therapy, and higher levels of CXCL13, a chemokine linked to B cell recruitment and renal function impairment. Importantly, CXCL13 levels were found to be positively correlated with the severity of tissue inflammation and the number of B cells in the renal tissue, highlighting its potential role as a marker for disease progression and treatment response in lupus nephritis patients.^439^

#### Sjögren syndrome

Sjögren’s syndrome (SS) is a chronic, systemic autoimmune disorder characterized by lymphocytic infiltration of the exocrine glands, primarily the salivary and lacrimal glands, and notable B-cell hyperactivity.^440^ Numerous studies have found the presence of TLSs in patients with Sjögren’s syndrome, which contribute to the progression of the disease. It was discovered that in salivary glands with TLSs, the expression of NCR3 (NKp30, CD337) was upregulated and showed a strong positive correlation with genes encoding granzyme B, perforin, and IFN-γ. This suggests an enhanced immune response in these regions. Furthermore, treatment with rituximab was found to prevent the reduction of NKp30 expression on NK cells, thus diminishing B cells, FDC networks, and ectopic germinal centers.^182^ In a separate evaluation focusing on the serum levels of CX3CL1 in primary Sjögren’s syndrome (pSS) patients, a significant elevation compared to controls was observed, indicating an inflammatory response. This elevation was also noted in patients with rheumatoid arthritis but not in those with Sicca syndrome, highlighting a potential marker for distinguishing between these conditions. Histological analysis further revealed the presence of CX3CL1 and its receptor within the salivary glands of pSS patients, specifically localized within tertiary lymphoid structures, reinforcing the role of this chemokine in the pathogenesis of pSS.^267^

#### Pemphigus

Pemphigus constitutes a group of rare autoimmune skin disorders characterized by the emergence of blisters and erosions on the skin and mucous membranes, resulting in significant pain and potential infections.^441^ In the study of patients with Pemphigus Vulgaris (PV) and Pemphigus Foliaceus (PF), it was found that CD3 T cells were present in all samples, and a high percentage contained CD20 B cells, CD138 plasma cells, HEVs positive for peripheral node addressin, and CD21^+^ monocytic cells, indicating that TLSs are prevalent in most pemphigus lesions.^292^ Similarly, another investigation into the skin of pemphigus patients revealed the presence of skin TLSs and desmoglein-specific B cells in chronic blisters. Within these TLSs, CD4 T cells, predominantly producing CXCL13, exhibited characteristics of activated Th1-like cells with downregulated genes related to T cell receptor-mediated signaling. Regulatory T cells (Tregs) were found to interact directly with CXCL13CD4 memory T cells, enhancing CXCL13 production through mechanisms involving IL-2 consumption and TGF-β stimulation. Additionally, the application of intralesional corticosteroids showed improvement in chronic blisters by reducing skin TLSs, suggesting a potential therapeutic strategy for pemphigus.^137^

#### Autoimmune encephalomyelitis

Autoimmune encephalomyelitis refers to a group of diseases caused by an autoimmune response, in which the body’s immune system mistakenly attacks and destroys tissues of the central nervous system (CNS), including the brain, spinal cord, and optic nerve. This type of inflammation is typically associated with the presence of specific autoantibodies that target particular antigens in the CNS.^442^ Research has illuminated the presence of TLSs within the meninges of multiple sclerosis (MS) patients, particularly during the progressive phase, which is tightly linked to cortical lesions and disability.^443^ Further investigations have revealed TLSs in the subarachnoid space, associated with extensive periventricular infiltration predominantly composed of CD20^+^ B cells and FDCs in some instances.^444^ Additionally, analyses of post-mortem brain tissues from MS cases have shown an upregulation in the expression of pro-inflammatory cytokines, such as tumor necrosis factor and interferon-gamma, within the meninges of patients with secondary progressive MS, characterized by the manifestation of TLSs. The severity of meningeal inflammation and the presence of TLSs in the MS brain is directly correlated with increased levels of TNF and IFN genes, along with their protein expressions in the meninges and cerebrospinal fluid compartments.^445^ This body of evidence underscores the significant role of TLSs in the pathogenesis and progression of MS, highlighting their potential as targets for therapeutic intervention.

#### Diabetes mellitus

Diabetes mellitus is a chronic metabolic disorder characterized by elevated blood sugar levels, primarily caused by insufficient insulin secretion or impaired insulin action. In a study involving 24 patients with type 1 diabetes, it was found that 21 of them exhibited insulitis in the pancreas, with 12 showing the presence of TLSs. The frequency of TLSs in the pancreas significantly diminished with increasing age. The occurrence of TLSs was closely associated with the severity of the disease, with the highest frequency observed in islets exhibiting insulitis (stages 1 and 2 islets). Mature TLSs, characterized by segregated T and B cell regions, were identified in only four patients with type 1 diabetes. Compared to patients with immature TLSs or without TLSs, those with mature TLSs had a significantly shorter duration of disease, with mean durations of 0.03 ± 0.035 years, 2.909 ± 2.709 years, and 2.948 ± 2.564 years, respectively. These findings suggest that the presence of TLSs in the pancreas of patients with type 1 diabetes is intricately linked to the progression and severity of the disease, and that mature TLSs may be associated with a shorter disease course.^446^

#### Uveitis

Uveitis is an inflammatory condition affecting the uvea, the middle layer of the eye, encompassing the iris, ciliary body, and choroid. It can lead to symptoms such as blurred vision, eye redness, pain, and photosensitivity, with severe cases potentially resulting in vision loss.^447^ The presence of TLSs was identified within the retinas of mice with uveitis, indicating a complex immunological involvement. In a related study on human samples, TLSs were found in 20% of the cases, particularly in those with chronic, persistent uveitis. This suggests that the condition leads to a significant dysregulation of ocular immune surveillance, marked by the emergence of TLSs.^291,448^ These findings collectively underscore the importance of TLSs in the pathophysiology of uveitis and highlight the critical role of immune system dysregulation in its progression.

#### Giant cell arteritis

Giant Cell Arteritis (GCA) is an autoimmune condition predominantly affecting individuals over the age of 50, characterized by chronic inflammation of large and medium-sized arteries, especially those in the head and neck region, potentially leading to vision loss and other severe complications.^449^ Recent investigations into GCA have revealed the pivotal role of TLSs in the disease’s pathology. In aortic tissues from patients with GCA, TLSs were identified in a significant majority, with a notable portion containing GCs, a feature less commonly observed in atherosclerotic tissues.^450^ Similar findings were echoed in temporal artery samples from GCA patients, where TLSs were frequently detected within the media layer, closely associated with high endothelial venules. These structures’ formation appeared to be linked to the expression of critical molecules and cells such as CXCL13, BAFF, APRIL, lymphotoxin β, and interleukins IL-17 and IL-7, among others. This association suggests a complex interplay of immune responses in GCA, further underscored by the observation that stimulating myofibroblasts with specific agonists and cytokines enhances the expression of BAFF and CXCL13.^51^ These insights underscore the intricate immunological landscape of GCA, highlighting the significance of TLSs in its pathogenesis.

#### Dermatomyositis

Dermatomyositis (DM) is an immune-mediated inflammatory disease characterized by specific lesions in the skin and skeletal muscles.^451^ It was observed that the lesions were infiltrated by a diverse immune cell population, including CD8^+^ cytotoxic T cells, macrophages, CD4^+^ cells, and B cells. Notably, these immune cells were arranged in a manner reminiscent of lymphoid follicles, with CD21^+^ follicular dendritic cells within these structures. Additionally, the vasculature associated with these lesions displayed features of HEVs, indicating a complex immune microenvironment within the affected muscle tissue.^452,453^ This indicates the presence of TLSs in DM, which influences the disease progression.

### Transplant

The history of solid organ and skin transplantation in experimental and clinical practice is marked by early attempts that were often thwarted by immune rejection.^454^ However, with advancements in more precise tissue matching and immunosuppressive techniques, the success rates of organ transplants have improved, although rejection remains a potential issue. Notably, the occurrence of rejection can lead to the formation of TLSs.

In the majority of current research on transplant-related TLSs, it was found that TLSs can enhance immune rejection, leading to a poorer prognosis.^455^ However, some studies have discovered that certain components within TLSs inhibit immune functions, facilitating immune tolerance, which may result in a reduction of immune rejection responses.^456^

#### Heart transplant

An analysis of 319 murine allograft heart transplants revealed the presence of TLSs in 78 cases, with a significant observation that 78% of allografts undergoing chronic rejection showed TLSs.^455^ Further validation highlighted the association of TLSs with immune rejection responses in allogeneic heart transplants. In another study, epicardial coronary arteries from 59 transplant patients were examined, showing a significant correlation between the presence and size of TLSs surrounding the coronary vessels and the time post-transplantation. Patients were divided into groups based on the average size of TLSs, revealing that those with larger TLSs (classified as TLS-2) had consistently shorter post-transplantation survival times compared to those with no or smaller TLSs (classified as TLS-0 and TLS-1).^62^ This suggests a strong link between the presence and size of TLSs and the progression of cardiac allograft vasculopathy.

#### Kidney transplant

In kidney transplantation, it has been discovered that although B cell subpopulations within TLSs include naive, plasma, and memory B cells predominantly localized within or near these organs, TLSs are associated with immune tolerance in the renal allograft model.^456^ The presence of small B cell clusters within the first two months post-transplantation is not linked to early rejection episodes. TLS-like structures can be present even in animals without rejected allografts and may disappear over time. While TLSs are more commonly found in samples with interstitial fibrosis and tubular atrophy (IFTA), their presence is also noted in samples without IFTA, indicating that the presence and density of clusters resembling TLSs reflect immune responses within the graft and do not necessarily predict poor graft outcomes or IFTA. TLSs are less common shortly after transplantation but become denser and significantly increase in rejected grafts over time. TLSs with GCs can be observed after 6 to 10 weeks post-transplantation, even in the absence of acute rejection episodes, and in acutely rejected grafts, the formation of TLSs can be detected as early as 12 days post-transplantation, suggesting a dynamic response in the graft immune environment.^457^

#### Lung transplant

IL-2 pretreatment of donors was found to effectively prevent acute immune rejection, as evidenced by macroscopic and histological examinations on day 5 post-transplantation. This intervention substantially enhanced graft survival, preserving lung functionality beyond 90 days. Moreover, the targeted deletion of FOXP3^+^ Tregs via diphtheria toxin receptor-mediated treatment disrupted the formation of TLSs, consequently eliciting a pronounced immune rejection.^458^

### Infection

During the transition to the chronic phase of inflammation, there is a gradual increase in the numbers of plasma cells and lymphocytes, which induces the formation of lymphatic vessels. Concurrently, blood vessels acquire the characteristics of

HEVs, specifically tailored to facilitate the extravasation of lymphocytes. In certain instances, chronic infiltration organizes into structured lesions, such as the granulomatous inflammation observed in tuberculosis, Crohn’s disease, and sarcoidosis. These chronic infiltrates form distinct aggregates rich in T and B cells, known as TLSs.^459,460^

#### Colitis

Large amounts of TLS are found in colitis lesions, which are formed by inflammation induction. Its formation process is closely related to the regulation of nerve secretion. In a colitis mouse model, the vagus nerve stimulates intestinal neurons, causing stromal cells to produce CXCL13, which in turn induces the formation of TLSs. However, cutting off the vagus nerve eliminates the formation of TLSs without affecting the progression of colitis, possibly due to the limitations of the model. This phenomenon is worth further study to clarify the functional relevance of TLSs in intestinal immune responses.^459^

Crohn’s disease is a specific inflammation of the intestines. Tissue imaging results show that the lymphatic structures of the patients with CD were dilated, and their permeability was altered by using Patent Blue Tracing technology. Compared to non-inflammatory bowel disease samples, there are more TLSs in Crohn’s disease samples. These TLSs reshape the structure of lymphatic pathways, altering lymph fluid flow and potentially affecting immune function and disease development.^370^ Guedj et al. found that the inflammatory cytokines TNF-α and LPS stimulate adipocytes around CD, making them organizers of TLS and thus coordinating TLS formation. They believe that in CD, TLSs are harmful because TLSs are associated with a more severe phenotype of CD.^460^

#### Chronic obstructive pulmonary disease (COPD)

New research evidence supports a key role for TLSs in exacerbating lung damage and promoting the exacerbation of COPD. Ladjemi et al. revealed that T cells activated B cells to secrete IgA by secreting IL-21, triggering an adaptive immune response. In COPD patients, increased pulmonary TLSs correlate with disease severity, but they lack direct evidence to show that TLSs will lead to the deterioration of COPD.^461^ Further research discovered that DCs were attracted by damaged airway mucosal SIgA immune barriers through the CCR2 pathway in COPD patients. This led to local lymphocyte aggregation, TLS formation, and T cell activation, thereby exacerbating lung damage.^179^ This study further reveals the association between the presence of TLSs and the pathological deterioration of COPD. And another study confirmed that IL-18^+^ myeloid cells in COPD lungs’ TLSs stimulate responsive lymphocytes to release IFN-γ, promoting tissue damage. IL-18^+^ macrophages and DCs are first located in pulmonary TLSs, making increased TLSs a marker of severe COPD.^462^ Thus, TLSs may exacerbate COPD tissue damage.

#### Nasal Polyps

In patients with chronic rhinosinusitis with nasal polyps (CRSwNP), TLSs are prevalent in the nasal mucosa. The TLSs contribute to the inflammatory burden in nasal polyps by synthesizing immunoglobulins, such as IgE and IgA, as well as proteins such as IL-1β and IL-33, thereby precipitating chronic conditions.^302,463–465^ More eosinophilic (accounting for 20.69%) and noneosinophilic (accounting for 17.31%) CRSwNP patients have TLSs, with higher levels of IgE, IgG, and IgA in eosinophilic polyps with TLSs, indicating increased inflammation. The study confirmed the presence of numerous plasma cells in TLSs that produce these immunoglobulins. Patients with TLSs have a longer disease course, more severe nasal congestion, and a higher frequency of past surgeries compared to those without TLSs.^464^

#### Bacterial infectious disease

TB is the most common bacterial infectious disease globally, and clinical results show that gender is an influential factor in the progression of TB. Further studies found that male patients may experience aggravated disease progression due to TLS impairment during Mycobacterium tuberculosis (Mtb) infection. By experimenting with Mtb infection in mice of both sexes, it was found that male mice had smaller B-cell follicles in the TLSs of the lungs, as well as lower expression of the chemokines CXCL13 and CCL19 in vivo, compared to female mice. This could be due to less IL-23 expression in males, impairing TLS formation in the lungs after Mtb infection, making males more susceptible to Mtb, which in turn promotes the progression of TB.^466^ In pneumococcal pneumonia, TLS’s role differs. In a model of continuous *Staphylococcus aureus* (*S. aureus*) infection in mouse lungs, B cells and T cells are key in TLS formation. Blocking TLS formation doesn’t cause death or increase bacterial load in the lungs, and the absence of B and/or T cells doesn’t exacerbate lung infection or reduce survival rate, indicating TLSs aren’t necessary in controlling S. aureus infection.^467^

Previous studies demonstrated that TLSs have a positive therapeutic role in viral infections. In models of pulmonary infection, TLSs rapidly develop following a viral or bacterial challenge, orchestrating a potent immune response. This response encompasses antigen-specific T and B cells, offering protection against harmful pathogens. By blocking the IL-1 signaling pathway, Neyt et al. found that iBALT formation was impaired, along with delayed viral clearance from the lungs, prolonged viral replication, and an increase in the severity of disease.^197^ Therefore, we can speculate that the formation of TLSs may be related to a good prognosis for viral infection. DCs are crucial for iBALT formation and maintenance in the lungs. The disintegration of iBALT by depleting DCs in the lungs confirmed that iBALT produces protective antiviral serum immunoglobulin antibodies through GC-dependent Ig class conversion.^177^ They play an important role in the removal of the virus. Therefore, iBALT plays a positive immune response role in viral infections.

#### Myocarditis

The formation of TLSs in recurrent severe myocarditis, potentially a case of autoimmune myocarditis, was first reported in 2020.^468^ The specific role of TLSs in this condition is unclear. However, in viral myocarditis, viral infection induces TLS formation in myocardial tissue, the amount of which positively correlates with the severity of the disease. Zhu et al. found the GC response of TLSs promotes local anti-heart autoantibodies (AHA) production, exacerbating heart inflammation. Blocking IL-17 or PDPN to reduce TLS formation can alleviate myocarditis symptoms.^469^ Therefore, TLS may become a new marker to predict the development and prognosis of viral myocarditis.

### Ageing

The frequency of TLS occurrence in aged individuals receiving stimuli significantly increased compared to younger individuals, which markedly influenced disease progression and prognosis. Yuki Sato et al. have established a potential new model for inducible sterile TLS formation, demonstrating the destruction of the adjacent renal parenchyma around the kidney TLS, a close association between TLS size and renal dysfunction, and the renal expression of the pro-inflammatory cytokine AID. In aged damaged kidneys, TLSs exhibit different stages of maturation. In the early stages of TLS formation, perivascular fibroblasts receive retinoic acid via paracrine signaling from Retinaldehyde Dehydrogenase fibroblasts and dedifferentiate into p75NTR fibroblasts, some of which acquire the ability to produce homeostatic chemokines (CXCL13/CCL19). As the TLT grows and expands, the number of retinoic acid-producing fibroblasts around the TLS gradually decreases. During this stage, CD21^+^ p75NTR^−^ FDCs (follicular dendritic cells) emerge as part of the stromal network, and PNAd^+^ HEVs (peripheral node addressin-positive high endothelial venules) develop within the TLS. Notably, TLSs are observed in aged, but not young, human kidneys, with cellular and molecular components highly similar to those in mouse TLSs. Therefore, age-dependent kidney TLS formation appears to be conserved across species, and the mechanisms underlying the progression of renal diseases in elderly humans might be similar to those in aged mice. Although renal TLSs have only been reported in patients with immune-mediated diseases such as autoimmune disorders, chronic rejection, glomerulonephritis, and infections, we found that age-dependent kidney TLSs are also present in various types of renal diseases, including diabetic nephropathy and benign nephrosclerosis. This suggests the universality of TLS formation with ageing.^371^

Studies have shown that as mice age, there are significant changes in the expression of immune-related genes in bladder tissue, particularly an increase in B cell-related gene expression, which is especially pronounced in female mice.^470^ The increased density of B cells in the bladder is closely associated with the formation of TLS, which are typically induced by exposure to commensal or pathogenic microorganisms, mucosal administration of IFN-1 activating vaccines, or chronic local inflammation.^471^ The formation of TLS in the bladder is not only linked to the elevated systemic levels of TNF-α observed during normal ageing but also to chronic inflammatory conditions such as interstitial cystitis and bladder cancer.^472,473^Mechanistically, the study found that with advancing age, there is a significant upregulation of B cell-related genes such as Cd19, Cd79a, Pax5, Mzb1, and Ms4a1 in the bladders of aged female mice. This suggests that the diversity and distribution of B cells in the bladder mucosa may be related to the formation of TLS. Additionally, the increased expression of CXCR5 and its ligand CXCL13 in the bladders of aged female mice further supports the recruitment of B cells to the bladder mucosa and the formation of TLS.^474^ The research also revealed an increased expression of immune checkpoint genes such as PD-L1 in the bladders of aged female mice, which may reflect a state of exhaustion in these immune cells. Furthermore, the expression of other immune checkpoint genes like Lag3 and Btla was significantly elevated in aged female mice, suggesting that these genes may play important roles in regulating immune responses. Since large-scale sequencing methods cannot identify the specific cell types exhibiting the exhaustion phenotype, further identification and functional analysis of these cells using single-cell sequencing and other methods will be crucial for comprehensively understanding these changes and developing precise therapeutic targets. Moreover, the study found that exposure to the carcinogen BBN led to increased PD-L1 protein expression in the bladders of aged female mice, particularly in urothelial and endothelial cells. This may be due to N-butyl-N-(4-hydroxybutyl) nitrosamine (BBN) -induced DNA damage and activation of the cellular IFN pathway.^475,476^ High expression of PD-L1 on endothelial cells is known to inhibit T cell activation, indicating that future research is needed to elucidate the mechanisms underlying this checkpoint to define its dynamic role in disease progression and potential immune evasion.^372^

### The relationship between TLSs and current anti-tumor treatment

**Fig. 6:**
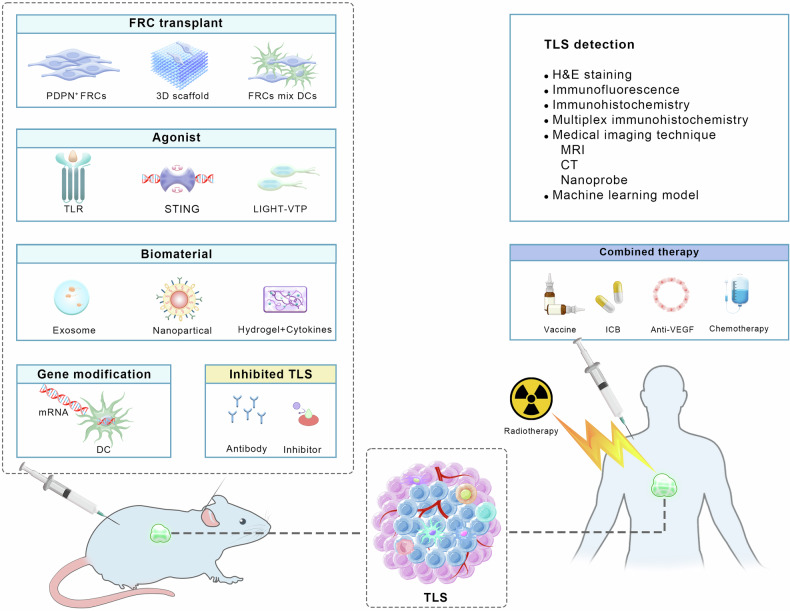
Therapies related to TLS formation and function. Several methods have been designed to induce TLS formation. Stromal cells injected alone or co-injected with artificial scaffolds, or DCs, provide a favorable space for TLSs. Chemokines and cytokines involved in TLS formation and B cell maturation, such as IL-13, CXCL13, LIGHT, LTα1β2, and CCL21, are introduced through engineering DCs and biomaterials. In addition, some receptor agonists also promote TLS formation. Other therapy strategies, including chemotherapy, vaccines, agonists, and ICB therapies, can also modulate TLS function

Research has demonstrated that TLSs are associated with the prognosis and responsiveness of various cancer treatments, such as surgical resection, immune checkpoint blockade (ICB), chemotherapy, antiangiogenic therapy, radiotherapy, and vaccines (Fig. 6).

In certain cancers, such as ESCC^396^ and colon cancer,^477^ TLSs have been identified as being related to the prognosis following resection. In a study involving 650 cases of surgically resected ESCC, it was demonstrated that ESCCs with mature TLSs exhibited significantly improved survival compared to both TLS-immature and TLS-negative ESCCs (*p* < 0.05). Specifically, the 5-year OS rates were 66.5%, 50.0%, and 50.2% (*p* = 0.001), while the disease-free survival (DFS) rates were 55.6%, 39.9%, and 46.3% (*p* = 0.003) for patients with TLS-mature, TLS-immature, and TLS-negative ESCCs, respectively.^396^ In the research findings on colorectal cancer, an analysis of the prognosis following resection of left and right colon cancers revealed heterogeneity in the presence of TLS. Specifically, the density of TLS serves as a prognostic marker for survival post-resection in patients with right-sided colon cancer. However, this marker does not offer a reliable prediction for patients with left-sided colon cancer.^477^

Neoadjuvant chemotherapy, a type of neoadjuvant therapy, was originally used as a preoperative treatment for tumors. However, in recent years, a new method of neoadjuvant chemotherapy has often been proposed: combination with other drugs. With the prevalence of immunotherapeutic agents, they are often combined with ICB in neoadjuvant chemotherapy, namely neoadjuvant chemoimmunotherapy. Many reports have described increased immune cell infiltration in tumors locally after treatment with neoadjuvant chemotherapeutic agents.^478^ In hepatoblastoma, pathological analysis revealed extensive TLSs in the tumor after neoadjuvant cisplatin chemotherapy, which were associated with a favorable prognosis.^479^ In the NEOSTAR trial, the combination of ipilimumab and nivolumab resulted in higher levels of intra-tumoral immune cell infiltration and more mature TLS characteristics.^480^ Additionally, the combination of nivolumab and cabozantinib increased the infiltration of effector T cells and memory T cells in hepatocellular carcinoma. And compared to those surgically resected, responders for combination treatment exhibit a highly immune-infiltrated focus, i.e., TLSs.^481^ Moreover, TLSs also play a prognostic role in neoadjuvant chemoimmunotherapy, such as NSCLC. Patients with mature or high-density TLSs show better prognoses in cancers after neoadjuvant chemoimmunotherapy.^397^ Sun and colleagues conducted a comparative analysis of tumor tissues with untreated, neoadjuvant chemoimmunotherapy, and neoadjuvant chemotherapy. Their investigation revealed increased CD8^+^ T cells and mature TLSs after neoadjuvant chemoimmunotherapy. Besides, the neoadjuvant chemoimmunotherapy group showed a higher major pathological response rate and pathological complete response rate.^397^ In another clinical study of NSCLC, preoperative application of 2-4 cycles of nivolumab combined with chemotherapy improved long-term survival, significantly increasing 3-year DFS from 50% to 63%. Responders also showed higher levels of mature TLSs.^482^ Xu et al. also reported that TLSs were more readily observed in patients responsive to neoadjuvant chemoimmunotherapy and validated the advantageous prognostic value of TLSs in NSCLC patients.^483^ These findings suggest that immunotherapy may induce TLS formation, and the maturity of TLSs could serve as a biomarker for better prognoses in patients with neoadjuvant chemoimmunotherapy. However, neoadjuvant chemotherapy treatment with combined corticosteroids impairs the formation of GCs in LSCC, negatively affects the development of TLS, and eliminates its prognostic value in patients with lung cancer.^5^

The presence of intra-tumoral TLS is associated with positive clinical outcomes and responses to immunotherapy in cancer. In multiple types of cancer, such as NSCLC,^398^ HNSCC,^484^ and ESCC,^383^ it has been discovered that TLSs enhance the efficacy of anti-PD-L1 therapy, increasing their responsiveness to ICB. In ESCC patients, mature TLSs serve as an independent prognostic factor. They show increased proliferative B cells, PCs, and memory B cells. Additionally, patients with mature TLSs and high T-cell infiltration (including CD8^+^ T cells, Th cells, and Th17 cells) exhibited higher survival rates. Notably, immunotherapy-treated patients had a higher proportion of mature TLSs compared to untreated patients (50.7% vs. 41.3%).^152^ Data from a study involving patients with triple-negative breast cancer suggests that TME with high levels of CCL19^+^ DC infiltration is characterized by immunogenic structures. After anti-PD-1 immunotherapy, CCL19^+^ DCs improved T-cell responses.^171^ Furthermore, high levels of CXCL13^+^ T cells and pro-inflammatory macrophages were associated with paclitaxel or its combination with anti-PD-L1 atezolizumab. And CXCL13^+^ T cells co-localized with B cells in TLSs.^485^ In uveal melanoma, pembrolizumab and entinostat treatment increased activated T cells, monocytes, and chemokines. And the presence of TLS prolonged PFS and OS in patients.^262^ Another study on metastatic melanoma demonstrated a significant improvement in survival trends for patients with tumor-infiltrating TLSs. In tumors lacking TLSs, T cells exhibited a dysfunctional molecular phenotype. Additionally, TLS^high^ tumors were linked to increased survival after treatment with CTLA4 antibody and anti-PD-1 antibody, either alone or in combination. Thus, TLS-associated genes can predict treatment responses to ICB therapy.^486^ The PANDORE clinical trial further supports the role of TLS in enhancing immunotherapy efficacy, showing that pembrolizumab preferentially targets Tfh cells in muscle-invasive bladder cancer, increasing the number of Tfh cells in TLSs. This promoted CD8^+^ T cell infiltration, thereby enhancing anti-tumor ability, which benefits patients.^487^ We speculate that if high-density TLSs persist in the tumor tissues, it may indicate that the patient’s immune system is still actively fighting against the remaining tumor cells. This could potentially decrease the risk of recurrence and extend both disease-free survival and overall survival. Conversely, low-density TLSs might indicate a poorer prognosis, as this suggests a weaker immune response to the tumor. Therefore, TLSs could serve as a potential prognostic biomarker for selecting individuals suitable for ICB treatment.

Furthermore, B cells might be essential for the antitumor effect. Generally, based on pan-B-cell phenotyping, B cells are associated with favorable clinical outcomes in many cancers. TLS^+^ tumors exhibited high frequencies of IgG-producing PCs and IgG-stained and apoptotic malignant cells, suggesting antitumor effector activity. Importantly, therapeutic responses and progression-free survival were correlated with IgG-stained tumor cells in RCC patients treated with immune checkpoint inhibitors. Thus, intra-tumoral TLSs sustain B cell maturation and antibody production, which is associated with response to immunotherapy, potentially via direct anti-tumor effects.^367^ In lung cancer, a cluster of genetic features, including CD20^+^ B cells, GC-B cells, memory B cells, and PCs, is highly expressed. Their density correlates with long-term survival.^59^ In ESCC, CD23 aggregates in TLSs. Compared to the low GC-TLS group, patients with high GC-TLS had a higher 3-year survival rate, especially after neoadjuvant chemotherapy.^488^ In patients with endometrial cancer, three major B-cell clusters in TLSs, including naive B-cell genes, GC-like genes, and plasma cell genes, were identified by scRNA-seq. Intrastromal CD20 cell density is associated with a lower risk of recurrence.^424^ The value of B cells and TLSs in the response to ICB has been demonstrated in several cancer types. Based on immune cell analysis, B cells were located in the tumor-stromal margins, which significantly enhanced the effect of anti-PD-1 therapy in melanoma patients.^86^ In another analysis of melanoma patients receiving nivolumab with or without ipilimumab as neoadjuvant therapy, responsive patients had higher densities of B cells and TLSs, as well as a higher ratio of TLSs to tumor area.^6^ Notably, prior to treatment, responding patients also exhibited higher B-cell and TLS densities. According to transcriptomics studies, responders exhibited greater intratumor memory B cells and fewer naive B cells, along with higher BCR diversity (both IgH and IgL).^6^

Radiotherapy triggers NK cell migration to the irradiated tumor by NF-κB and mTOR-coordinated irradiated tumor cells to secrete chemokines.^489^ In addition, local radiation therapy induces increased expression of MHC-II molecules as well as death receptors FAS/CD95 and DR5 on tumor cell surfaces, and synergies with antigen vaccines.^490^ A study examined tissue samples from 198 patients who had undergone surgery and found that TLSs are associated with a favorable prognosis for lung adenocarcinoma (LUAD). The research also revealed that low-dose radiation therapy (LDRT) promoted the early formation of TLSs in a Kras-LSL-G12D mouse model of lung cancer. Furthermore, the combination of LDRT and anti-PD-1 therapy significantly enhanced the maturation of TLSs in mice with LUAD, resulting in a more robust anti-tumor response. This antitumor effect was closely correlated with the number of CD8^+^ T cells within the TLSs.^491^

Human tumor antigens have low immunogenicity, and now the research and development of cancer vaccines have been explored by many scholars. The formation of TLS was also observed when therapeutic vaccination was administered to patients with immunocompromised tumors. This indicates that TLS in the tumor can be induced by the cancer vaccine and is associated with increased immune function and beneficial clinical outcomes.^492–494^ Therefore, adjuvants can be added to support the formation of TLS induced by cancer vaccines. However, most tumor vaccines are still experimental, and only a few are approved.

## The detection of TLS

TLSs are associated with disease development and prognosis. Given their potential as prognostic markers, efficient detection methods are needed. We will summarize some techniques for detecting TLSs and aim to provide valuable insights for research and clinical application (Fig. 6).

### Hematoxylin and Eosin staining

Hematoxylin and eosin (H&E) staining of tissue sections allows for the evaluation of TLS size, density, and maturity, including the formation of lymphocyte clusters and GC.^5,19,495^ HEV-like features can also be observed in TLSs.^496^ Currently, H&E staining is widely used for detecting TLSs due to its simplicity and cost-effectiveness. However, it provides limited information and allows only for rough quantification.^497,498^ At the same time, because of objective and subjective differences between observers, there may be inconsistencies in the results.^497^

### Immunohistochemistry (IHC)/immunofluorescence (IF) techniques

Immunohistochemistry/immunofluorescence (IHC/IF) techniques can label 3–4 markers in tissue slices, providing a basic cell phenotype analysis of TLSs. In contrast, the H&E staining technique can typically label 1–2 markers.^378^ These techniques can clearly identify the CD20^+^ GC and determine its positional relationship with TLSs in tumor tissue.^404^ Additionally, they can reveal the PDPN^+^FAP^+^DAPI^+^ fibroblast network and CD3^+^CD26L^+^ T cells in TLSs.^41,170^ Cell markers and chemokines, such as CD3, CD19, CXCL13, and CXCL21, were detected in TLSs through ICH/IF.^41^ Although this technique has advanced in the number of markers, the precise and detailed visualization of TLS structure and comprehensive research still require the combined use of multiple tissue slices.^41,378^

### Multiplex immunohistochemistry (mIHC)

To overcome the limitations of IHC/IF, mIHC technology allows for the labeling of more than 5 markers to comprehensively analyze the structure of TLSs in a single tissue section. Compared to IHC/IF with multiple slices, mIHC increases the credibility of the results. With the support of mIHC, researchers inferred the composition and function of TLSs more accurately.^378,499^ Helmink et al. used mIHC to analyze the TLS structure, clarifying CD20^+^ B cells co-localized with CD21^+^ FDCs, CD4^+^ T cells, CD8^+^ T cells, and FOXP3^+^ Treg cells in GC, in proximity to MECA79^+^ HEVs.^6^ Stowman et al. tilized six antigens—CD20, CD8, PNAd, Ki67, CD83, and FOXP3—to detect TLS structure. They found that CD20^+^ B cell follicles are located in the center, surrounded by CD8^+^ T cells and PNAd^+^ vascular systems, and CD83^+^ DCs, FoxP3^+^ Treg cells, and Ki67^+^ proliferating cells.^500^ The Opal^TM^-TSA mIHC system provides a standardized experimental protocol for detecting TLS characterization, capable of simultaneously label 7 markers, including CD4, CD8, CD19, CD21, DC-LAMP, PNAd, and DAPI.^501^

### Medical imaging technigue

While H&E staining, IF, IHC, and mIHC are invasive methods for detecting TLSs, medical imaging technologies, such as computed tomography (CT), positron emission tomography (PET), single-photon emission computed tomography (SPECT), and magnetic resonance imaging (MRI), provide non-invasive and real-time alternatives. For instance, CT images of lung adenocarcinoma patients revealed part-solid nodules (PSNs). Comparing with the pathological results, the punctate solid changes in PSN might be TLSs.^502^ However, CT alone only proves the aggregation of lymphocytes, lacking specific evidence to support the existence of TLSs. Dorraji et al. injected ^99m^TC-labeled albumin Nanocoll (^99m^TC-Nanocoll), showing TLSs in the kidneys and pancreas during the progression of systemic lupus erythematosus via SPECT. They found that TLSs were located near the islets in the pancreas.^503^ However, ^99m^TC-Nanocoll is taken up by cells through the phagocytosis receptor rather than specific binding with TLS-related markers. Although lymphocytes show a high signal of ^99m^TC-Nanocoll, it still lacks TLS specificity.^503^ Due to limitations of accuracy and specificity, few imaging applications are used for direct TLS detection. Researchers have developed materials for imaging single cells in TLSs. For example, a zirconium-89 labeled deferoxamine-modified anti-CD3 mAb detected CD3^+^ T cell infiltration in tumors via PET.^504^ Similarly, 89Zr-labeled PEGylated single-domain antibody fragments were used for PET imaging of CD8^+^ T cells in and around tumors.^505^ Thorek et al. monitored the distribution and consumption of B cells labeled with superparamagnetic iron oxide nanoparticles and near-infrared fluorescent dyes through MRI in the spleen of mice.^505^ However, we believe that the imaging of a single lymphocyte cannot effectively indicate TLS presence.

### Machine learning model

Machine learning techniques are being used to improve the efficiency and accuracy of TLS evaluation in histopathological sections. Researchers have successfully achieved automatic TLS identification in H&E sections by combing the DeepLab v3^+^ network, active contour models, and lymphocyte segmentation methods. This method shows a specificity of 92.87% at a sensitivity of 95% and a specificity of 84.32% at a sensitivity of 99% in the detection of TLSs.^506^ The HookNet model has been proposed for the semantic segmentation of whole slide images. Its further version, the HookNet-TLS model, allows for TLS quantification in H&E digital pathology slides, and the accuracy is comparable to that of senior pathologists.^507,508^ Additionally, an interpretable machine learning model can automatically detect and quantify TLSs, classify them based on their status, and evaluate their prognostic value.^497^ These advances are crucial to enhancing our understanding and utilization of TLSs.

## Strategies for targeted regulation of TLS

### Inducing the formation of TLS

#### Engineered DCs

The coexistence of mature DCs and CD8^+^ T cells is a key prognostic factor for overall survival, and DCs are usually associated with TLSs.^509,510^ Thus, DCs are potential targets for inducing TLS formation (Fig. 6). By genetically engineering DCs, such as creating DC-Ad-CCL21 via adenovirus vectors, it is possible to increase CD8^+^ T cell infiltration at tumor sites, enhance T cell immunity and elevate PD-L1 mRNA expression in mouse bronchoalveolar cell carcinoma.^511^ This complements ICB therapies to ensure the effectiveness of anti-tumor immunotherapy. In a phase I trial on NSCLC patients, Ad-CCL21-DC increased the number of CD8^+^ T cells by an average of 3.4 times by intra-tumoral injection in over half of the patients.^512^ Tumor immunosuppression, particularly immune resistance against tumor-specific T cells, is associated with dysregulated expression of tumor immune checkpoint proteins. The above two studies targeting engineered DCs enhanced antigen presentation and promoted tumor T-cell infiltration and T-cell reactivity. It can increase PD-L1 expression while upregulating tumor co-stimulatory molecules. This further enhanced the anti-tumor effect. However, the role relationship between TLSs induced by DC and anti-tumor immune checkpoint inhibition needs to be further explored.^511–513^ In MCA205 sarcoma and MC38 colon cancer, T-bet gene-transduced DCs (mDC.T-bet) slow tumor growth, increase lymphocyte infiltration, and promote TLS development.^514^ Overexpression of IL-36γ in DCs also promotes rapid lymphocyte infiltration and TLS formation in MCA205 tumors in mice. The co-injection with rmIL-1F5, an IL-36R antagonist, suppresses the anti-tumor response.^515^ Furthermore, a CD1c^+^ DC vaccine increased CD8^+^ T cell activity, showing promising immune responses in metastatic melanoma patients.^513^ Ad-IL-15 vaccine induces DC maturation and activates the STING pathway, enhancing CD8^+^ T cell function against tumors.^516^ Here, we further summarize and discuss how engineered DCs as vaccines can effectively induce TLSs and suggest their role in upregulating PD-L1 expression. However, there is no clear experimental data to confirm the relationship and regulatory mechanism between them.

#### Stromal cell transplantation

The activation of fibroblasts initiates TLS formation by providing space and cytokines.^517^ Researchers have extensively studied stromal cells for their potential to induce TLSs. (Fig. 6) Zhu et al. demonstrated that PDPN^+^CD31^−^ FRCs extracted from lymph nodes were transplanted into the subcutaneous. After 3–4 months, significant swelling was observed at the injection site, with an increase in CD69^+^ PD-1^+^ T cells, indicating TLS formation.^518^ The expression of IFN-γ was detected in TLSs, which inhibit tumor growth and reduce PD-1 and TIM-3 expression on T cells.^518^ This suggests that transplanting stromal cells can induce the formation of functional TLSs. However, direct injection of stromal cells may not form sufficient or functional TLSs in a short time, as they must provide space for TLS formation in advance.^519^ The interaction between local fibroblasts and DCs can modify the matrix characteristics, supporting TLS formation.^520^ Lee et al. constituted a 3D fibrous spheroidal scaffold loading stromal vascular fraction cells from adipose tissue and/or mature DCs. This scaffold provides a specialized niche for DCs, promoting T cell recruitment and T cell lymph cluster formation in situ, thereby enhancing antitumor effects. Therefore, co-injecting DCs and stromal cells significantly improves TLS formation efficiency and success rate.^521^ In a previous study, researchers used TEL-2 thymic stromal cells with normal immune function as a substitute for lymphoid stromal cells. They engineered TEL-2 cells to express LTα (TEL-2-LTa) and devised a sponge-like 3D scaffold made of bovine collagen to load these cells. After transplanting the mixture into the subcapsular space of the mice’s kidneys, they observed B-cell clusters, T-cell clusters, and HEVs by immunostaining, with the formation of FDC networks and GC, which are highly similar to SLO structures.^522,523^

#### Recombinant cytokines

As previously discussed, numerous cytokines are involved in TLS neogenesis, and their injection has been used to modulate TLSs (Fig. 6). For example, CXCL13 and CCL21 recruit lymphocytes by binding to CXCR5 and CCR7 respectively, which is related to TLS formation.^524^ In a mouse ovarian cancer model, intraperitoneal injection of recombinant CXCL13 increased intra-tumor TLSs. Concurrently, the infiltration of CD8^+^ T cells around TLSs increased, aiding in TLS formation and improving survival rates.^254^ Intra-tumoral injection of CXCL13/CCL21 promoted lymphatic infiltration and TLS formation in a mouse model of PDAC. Notably, co-administration of CXCL13/CCL21 with gemcitabine into orthotopic tumors significantly increased the efficiency of TLS formation, resulting in tumor reduction.^525^ Moreover, Treg cells, a type of T cell subtype, are closely related to immunosuppression in TLSs. FOXP3^+^ Treg cells increased after treatment with IL-2/anti-IL-2 mAb complexes (IL-2cx) within TLS in allogeneic lung transplants of mice, facilitating allograft acceptance and increasing the survival time over 90 days.^458^ In contrast, the absence of IL-2cx or the depletion of FOXP3^+^ Treg cells inhibited TLS development.

LIGHT normalizes intra-tumoral blood vessels, promoting TLS formation. Yukun Huang et al. co-loaded α-mangostin and plasmid encoding LIGHT into Nano-sapper, which reverses the abnormally activated fibroblasts, normalizes tumor vessels, promotes T cell infiltration, and ultimately induces TLS formation.^526^ CGKRK is a short peptide that targets adhesion molecules on the tumor vessel in glioblastoma. The LIGHT-CGKRK protein, which consists of CGKRK and LIGHT, is produced by transfecting E. coli with the pET-44a plasmid. In a glioblastoma model, it induces HEV formation in the tumor and enhances cytotoxic T cell entry following intravenous injection.^239^ Researchers have also engineered chimeric antigen receptor (CAR) T cells (Pbbz-LV CAR-T) to express PSMA and LIGHT, and OT-1 T cells (LIGHT-OT-1) to overexpress LIGHT. In a mouse tumor model injected with Pbbz-LV CAR-T, the antitumor effect was enhanced. In the B16F10-OVA melanoma tumor model injected with LIGHT-OT-1 cells, normalized tumor blood vessels and intra-tumoral lymphatic structures were observed.^238^ While none of the studies directly detected TLSs, vascular normalization and HEV formation are conducive to TLSs, suggesting that LIGHT is an effective target in regulating TLS formation and anti-tumor effects.

#### Toll-like receptors agonists

TLRs, part of the pattern recognition receptor family, regulate immune responses and are linked to autoimmune diseases and TLS formation.^527^ (Fig. 6) Li et al. found that monocytes activated by LPS (a TLR4 agonist) generated TGF-β1 stimulation, inducing the activation of Sox4 in CD4^+^ T cells in nasopharyngeal carcinoma. This led to the maturation and proliferation of PD-1^+^CXCR5^-^CD4^+^ Th-CXCL13 cell, which secrete CXCL13 and facilitate TLS occurrence.^255^ In a myasthenia gravis mouse model, TLR4 activation maintained high CXCL13 mRNA expression induced by Poly(I:C) in the thymus, leading to B cell recruitment related to GC development.^528^ Another study on PEMBROSARC found that intra-tumoral administration of G100, a TLR4 agonist, increased CD4^+^ T cell and CD8^+^ T cell infiltration in soft tissue sarcoma patients.^529^ However, its effect on TLS formation is unclear due to the limitations of objective factors in this study. TLR7 is also associated with TLS formation. In a chronic hepatitis B chimpanzee model, Li et al. observed that lymphoid aggregates formed in the portal areas of the liver after administration of GS-9620 (a TLR7 agonist). These aggregates, composed of CD3^+^ T cells, CD8^+^ T cells, and B cells, appeared to be immature TLSs, as they had not formed GCs.^530^ Despite these findings, the effect of TLR agonists on TLS formation requires further confirmation.

#### Lymphotoxin β receptor agonists

Stimulation of LTβR with polyclonal antibodies enhanced the construction of FRC networks, which is crucial for TLS^531^ (Fig. 6). In CT26 colon cancer mice, an agonistic anti-murine LTβR monoclonal antibody ACH6 increased the infiltration of T cells and B cells, inhibiting tumor growth. However, they observed only an increase in T cells in the tumor, which could be a precursor to the development of TLS.^532^ Since the initial stage of TLS formation is characterized by preferential infiltration of T cells, followed by the accumulation of B cells. Another study confirmed that LTβR agonists increased HEV density in PyMT-BC and RT2-PNET models by 1-fold and 4-fold, respectively, with lymphocyte infiltration.^282^ Furthermore, anti-LTβR monoclonal antibody agonists can also reverse the disorder of DC subsets in LTβ/LIGHT-deficient mice and promote DC cell proliferation, differentiation, and migration.^533^ While none of these studies directly detected TLS formation, they provide ideas for future exploration.

#### STING agonists

Stimulator of interferon genes (STING), a pattern recognition receptor, recognizes the non-self DNA from viruses or tumor cells and activates DCs or ECs to secrete cytokines, promoting tumor blood vessel normalization (VN) and lymphocyte infiltration. (Fig. 6) Studies have confirmed that low-dose STING agonists, such as cGAMP and ADU-S100, can be used as a method to mediate tumor immunity.^534^ In a Lewis lung cancer model, intra-tumoral injection of ADU-S100 promoted VN, modulating the tumor immune microenvironment, and increasing CD8^+^ T cell infiltration, controlling tumor growth.^535^ Additionally, ADU-S100 administration induced the secretion of cytokines and chemokines, including LTα, LTβ, LIGHT, CCL19, and CCL21, as well as the anti-angiogenic factors Tnfsf15 and Cxcl10, in a mouse melanoma model. This induced tumor blood VN, enhanced CD8^+^ T cell and DC infiltration, and non-classical TLS neogenesis.^536^ Demaria et al. found that using the dinucleotide cGAMP to activate the STING pathway increased the number of CD8^+^ T cells and inhibited tumor growth, with endothelial cells identified as the main responders to STING activation.^537^ Additionally, in Influenza A virus-infected IFN-αr1^−/−^ mice, CXCL13 mRNA decreased on the third-day post-infection, but its expression increased after injecting IFN-β in the lungs of wild-type mice. And co-administration of IFN-β and cGAMP can induce the formation of GC in the lungs.^268^ However, STING agonists alone cannot recruit B cells, suggesting the need for combination therapies to enhance the antitumor effect of TLSs.

#### Nanoparticles

Nanoparticles commonly used as transport carriers for small-molecule drugs targeting tumors, have shown promise in inducing TLS formation. (Fig. 6) In the B16F10 melanoma model in mice, researchers synthesized a novel type of lipid nanoparticle (LNPs), characterized by diamino-ionizable lipid materials with various head groups (DAL). These nanoparticles encapsulated mRNA-encoding cytokines, including IL-12 and IL-27. Following intra-tumoral delivery, robust immune effector cell infiltration was observed in the tumor tissue. Notably, they also found that DAL4-LNP selectively delivers mRNA to CD19^+^ B cells rather than T cells. The combination of DAL4-LNP-IL-12 mRNA and DAL4-LNP-IL-27 mRNA worked synergistically to inhibit tumor growth without obvious toxicity.^538^ However, the formation of TLSs was not assessed, making it challenging to ascertain the efficacy of this approach in promoting TLS development. Wiley et al. developed the protein cage nanoparticle (PCN). This PCN is a hollow spherical protein cage structure with a diameter of 12 nm prepared from the small heat-shock protein (sHsp 16.5) of the hyperthermophilic archaeon Methanococcus jannaschii. Treatment with PCNs successfully induces the formation of iBALT structures in the submucosa of the lung, comprising CD20^+^ B cells, CD4^+^ T cells, FDCs, and CD8^+^ T cells after treatment. Mice infected with the influenza virus showed higher survival rates, faster virus clearance, quicker virus-specific antibody production, and reduced lung injury.^539^ Furthermore, FHK-pLIGHT@CaMP, known as Nano-sapper, composed of antifibrotic phosphates modified by α-mangostin and plasmids encoding LIGHT, was administered in a mouse PDAC model. It targets tumor tissue with the ECM glycoprotein (tenascin C) targeting peptide (FHK); subsequently, MP and LIGHT cooperatively improved vascular structure and promoted CLT cell infiltration. The histological results showed a clear TLS structure.^526^ Although these methods are still limited in regulating TLSs, they provide valuable insights into the rational utilization of nanoparticles for potential TLS induction and modulation.

#### Hydrogel

Hydrogel, a multifunctional platform, can bind and retain water, forming a viscoelastic gel polymer network in an aqueous solution. Due to these properties, they are frequently utilized in various biomedical applications, such as carriers for the localized release of antitumor drugs.^540^ (Fig. 6) Kobayashi et al. created a collagen sponge scaffold containing slow-release hydrogel beads that load LTα1β2, CCL19, CCL21, CXCL12, CXCL13, and soluble RANK ligand. When transplanted into the subrenal capsule space of Balb/c mice, it induced a TLS-like structure surrounded by lymphatic capillaries.^541^ Another study developed a STING-activated hydrogel (ZCCG), composed of Zn2^+^ and 4,5-imidazole dicarboxylic acid complexes, integrating chitosan nanoparticles and CpG. When administered intra-tumorally in melanoma mice, ZCCG activated the cGAS-STING and TLR9 pathways, inducing the secretion of chemokines CXCL13, CCL19, and CCL21, promoting DC maturation, enhancing lymphocyte infiltration, producing pro-inflammatory cytokines, and inducing TLS formation in the tumor, ultimately inhibiting tumor growth.^542^ Additionally, a potent anti-tumor nanoscale peptide hydrogel (MRM-coated spores) was reported to increase CXCL9 expression, promote CD8^+^ T cell responses, and induce M1 macrophage polarization. This hydrogel coated C-novyispores with a melittin-RADA32 (MR) hybrid peptide and loaded metformin (MET). It enhances the antitumor immune activation of C-novyi-NT spores, remodeling the immune system by slowly releasing MET after intra-tumoral injection.^543^ However, its effect on inducing the formation of TLSs needs further study.

#### Biological scaffold

Synthetic scaffolds, with their porous structure, can induce the formation of TLSs by providing necessary space and releasing encapsulated cells or cytokines.^544^ (Fig. 6) Tomei et al. created a macroporous polyurethane scaffold using type I collagen and Matrigel. In vitro, they cultured FRCs within the synthetic scaffold, and finally constructed a 3D lymph node T-zone stromal cell network tissue model. This 3D scaffold with FRCs not only provides a space for lymphocyte aggregation but also secretes the chemokines CCL19 and CCL21 to enhance lymphocyte migration and localization.^545^ Stachowiak et al. developed a composite material that mixed macroporous polyethylene glycol (PEG) hydrogel and collagen matrix. The PEG hydrogels can stably retain CCL21, while the collagen matrix loads T cells and DCs.^546^ Recently, a 3D-printed scaffold loaded with immunomodulators was developed as a vaccine. Its porous structure, similar to lymph node structure, facilitated immune cell infiltration and TLS formation. The scaffold recruited APCs and T cells in vivo and trained DCs and naive cells to mature. This 3D scaffold inhibits metastatic tumor development and prolongs survival time in tumor resection and metastasis models.^547^ However, the exploration of synthetic scaffolds to induce TLS formation remains challenging, with safety and feasibility issues limiting their development.

#### Exosomes

Apoptotic extracellular vesicle-like exosomes (ApoExo) are a special type of extracellular vesicle with a diameter of 30–100 nm, belonging to the structure of nanoscale lipid encapsulation. These vesicles are released by endothelial cells activated by caspase-3. ApoExo contains an active 20S proteasome core, basement membrane protein glycan LG3 C-terminal fragment, and long non-coding RNA. Among them, the active 20S proteasome core can regulate immunogenic activity.^548–550^ The secretion of ApoExo increased the number of GC-B cells and Tfh cells and promoted the generation of LG3 IgG in mice, exacerbating the rejection reaction.^550^ In aortic transplanted mice, the formation of TLSs was observed after ApoExo injection into the graft, accompanied by massive γδT cell infiltration, promoting IL-17 expression, resulting in complement deposition, and autoantibody production.^549^ This suggests that exosomes are a feasible approach to regulating TLSs. (Fig. 6).

### Inhibiting the formation of TLS

#### Antibody-based blockade

Th17 cells play a crucial role in the development of TLSs by secreting cytokines, such as IL-17 and IL-22.^147,551^ IL-17A^+^ PDPN^+^ T cells are present within TLSs in the myocardial tissue of viral myocarditis. Targeting Th17 cells by using IL-17 mAb and PDPN mAb inhibits their proliferation and differentiation, therapy decreasing pro-inflammatory factor secretion. This reduction lowers reduces the number of active B cells and the formation of TLSs.^469^ In ischemic reperfusion injury (IRI) kidney injury models, the absence of IL-17A results in reduced TLS volume in IL-17A^−/−^ mice. Although IL-17A^−/−^ mice exhibit similar cellular components in TLSs as seen in normal mouse IRI models, the proportions of B220^+^ B cells, CD3^+^ T cells, CD4^+^ T cells, CD8^+^ T cells, and FRCs are significantly reduced in IL-17A^-/-^ mice, leading to impaired TLS function.^219^

In a Sjögren’s syndrome model, Th17 cells expanded in Il-27ra^−/−^ mice. Using anti-IL-17A antibodies reduces infiltration of T cells and B cells in salivary glands, along with impaired GC function.^207^ Additionally, the IL-27 antibodies increase CD21^+^ FDC networks and GL7^+^ GC B cells in IL-27ra^−/−^ mice. This suggests that IL-27 negatively regulates the GC response in TLSs. Notably, IL-17 inhibitors can reverse the abnormal activation of TLSs caused by the lack of the IL-27 signal.^207^ Liu et al. found that delivering IL-12 and IL-27 mRNA promoted immune effector cell infiltration, mainly CD8^+^ T cells and NK cells, and significantly inhibited tumor growth.^538^ This result is the synergy of IL-12 and IL-27 and does not appear to conflict with the role of IL-27 in inhibiting the GC response in TLSs.

Tfh cells secrete CXCL13 to recruit and activate B cells, mediating TLS formation.^390,408,409^ In chronic kidney disease, targeting Tfh cells reduces IL-21 expression by blocking the ICOS signal, which inhibits TLS formation and alleviates renal fibrosis.^225^ Additionally, Treg cells appear to promote TLS formation indirectly. In a mouse model of fibrosarcoma, after Treg cell depletion, activated CD8^+^ T cells promote the formation of HEVs by injecting anti-CD4 and anti-CD8 antibodies. This promotes T-cell infiltration and tumor destruction.^136^ Moreover, in SS-NOD mice, the B-cell zone and T-cell zone decrease, the FDC network is lost, and HEVs are reduced after blocking LTβR by LTβR-Ig in the salivary glands. Meanwhile, the function of salivary glands, which was lost due to TLS, has been partially restored.^552^ In a rheumatoid arthritis mouse model, knocking out CXCR5 and CCR7 reduced mature TLSs and joint destruction.^553^ Notably, blocking CXCL13 in NOD mice destroyed TLSs, but it didn’t decrease B cell infiltration and inflammation^554^ (Fig. 6).

#### Inhibitors

AY61-3606, an inhibitor targeting spleen tyrosine kinase, reduces B cell vitality in tonsils, impairing the GC response.^554^ However, there is no research to prove that BAY61-3606 can affect the formation of TLSs. In contrast, FTY720, a sphingosine-1-phosphate receptor regulator, decreases B220^+^ B cells and CD4^+^ T cells in the kidney, thereby reducing TLS volume.^219^ Interestingly, FTY720 can prevent diabetes by maintaining TLS integrity in the pancreas of NOD mice^555^ (Fig. 6).

## Perspective and future challenges

Tertiary lymphoid structures (TLSs) are observed in various diseases, and with a complex association with prognosis. Some studies suggest TLSs may promote tumor progression,^495,556^ which contradicts findings that associate TLSs with improved patient survival. This discrepancy is likely due to the heterogeneity of TLSs, which complicates their clinical application. Recent reports highlight significant differences in the morphology, composition, and GC responses within TLSs.^44,557^ These differences can affect the TME through various inflammatory cytokines, leading to a switch in humoral immune type in TLSs, which will severely promote tumor progression.^557^ Additionally, the characteristics of TLSs are different between the early and late stages of disease.^44,234^ And the maturity of TLSs also influences the risk of tumor recurrence.^19^ Therefore, given the “double-edged sword” nature of TLSs, it is important to consider the proportion of various immune cell subpopulations within TLSs, especially the diversity of immune cell phenotypes. The TME regulates different immune cell subtypes and their phenotypes, and a detailed characterization of these subtypes in TLSs will help understand their impact on disease and their contribution to TLS formation and function. Moreover, the specific aspects of TLS formation, particularly the involved cell signaling pathways, need further exploration.

Currently, tumor immunity research focuses primarily on T cells, with B-cell immunity being less understood. Since TLSs are characterized by lymphocyte aggregates centered on B cells, future research should shift towards understanding B-cell involvement in tumor immunity. The activation of GC-B cells and antibody secretion in TLSs emphasize the importance of B cells in the immune response. In addition, it remains unclear whether the varying locations of TLSs lead to differences in their composition, immune responses, or tumor progression. For example, recent studies have shown that intra-tumoral TLSs are associated with prolonged patient survival in HCC, whereas peritumoral TLSs have no impact on prognosis and may even promote tumor progression.^19,385^ Notably, TLSs that negatively regulate disease often appear in the peritumoral region, regardless of their maturity. However, peritumoral TLSs are not always associated with a poor prognosis, as they can still support patient survival.^558^ The various prognostic implications of TLSs are due to discrepancies in detection methodologies in cancer. Considering that the density, localization, and maturation stage of TLSs are pivotal determinants of their prognostic value, it is crucial to stratify data collection and analysis according to these criteria. This approach will significantly improve the precision of prognostic estimations.

Given the diversity and complexity of TLS structures, various methods have been developed for detecting them, including IHC/IF, H&E staining, and transcriptional analysis.^559^ However, these traditional methods are invasive, and limited by subjective or objective factors, such as standardized differences in evaluators and information loss during sample processing, which can distort the real data of TLSs. This limits their application in disease prediction and treatment. Therefore, the development of a reliable method for detecting TLS formation, maturation, and density is essential for maximizing the clinical benefits of TLSs. While computerized scanning techniques and magnetic resonance imaging (MRI) have been used for TLS detection, they lack the specificity and accuracy to distinguish between TLSs and high-density lymphocyte aggregates.^503^ Biomaterials provide a more accurate and sensitive way to detect TLS components. They are typically designed as fluorescent imaging probes that chelate antibodies and facilitate radiolabeling for targeted biomarkers.^135^ However, current nanofluorescent probes are insufficient for simultaneously detecting more than two components of TLSs, let alone displaying their structures, which is important for defining TLSs.^560^ Accordingly, imaging techniques have significant limitations in monitoring TLSs. Furthermore, a big data learning model has been proposed for accurate digital quantitative analysis, which translates TLS histopathological features into a set of universal and reproducible mathematical values to standardize TLSs.^497^ However, the method requires a large number of identified TLS-containing tumor sections for algorithmic practice, which is a lengthy process, and it also relies on the previously accurate identification of TLSs. And it can only identify TLSs in tissue sections and cannot monitor TLS formation in real time, providing limited value for disease treatment. There is still a lack of a standard method for detecting TLSs, which has led to a significant reduction in confidence in the results across different studies. Merely identifying the cellular components of TLSs is not sufficient to accurately determine their presence, localization, and maturation stage. Given the significant role of TLSs in various diseases, particularly their potential to enhance immunotherapy, there is an urgent need to develop a minimally invasive or non-invasive method for monitoring TLSs. This will create new possibilities for the development of future patient treatment programs. Until now, only techniques such as multiplex immunohistochemistry (mIHC) and multiplex immunofluorescence could elucidate the spatial structure of TLSs. And mIHC is still the most accurate way to identify TLSs, as it labels the major cellular components in TLSs and reveals their structure. Importantly, because the heterogeneity and maturity of TLSs have different effects on disease, it is necessary to mark CD21^+^ FDCs and CD23^+^ GCs as TLS indicators. This will provide a detailed delineation of the potential relationship between TLS and disease, particularly the differences in maturity and components. At the same time, it will greatly enhance the clinical guidance value of TLSs and provide a measure for the extent of targeted regulation of TLSs in the future.

TLSs have shown great potential as a new field of immunotherapy. Future studies should focus on the relationship between TLSs, disease risks, and clinical outcomes. Recent articles have demonstrated the value of TLSs as a prognostic indicator of disease, particularly cancer.^561^ This may identify TLSs as a new biomarker for clinical intervention, contributing to the development of more effective disease prevention and diagnostic methods. Furthermore, TLSs have attracted interest as potential anti-tumor immune mediators. Therefore, it is a promising research direction that explores a combined therapeutic strategy based on TLS modulation to improve clinical outcomes. Modulating TLS immune capacity through targeted delivery of specific agonists, antibodies, or therapeutic agents holds immense promise for enhancing the efficacy of biotherapeutics. However, given their complexity and time-consuming features, it is difficult to obtain the desired results with a single approach. The lack of appropriate induction protocols, imaging methods, and research models has also hindered the scientific research and clinical application of TLSs. Studies have shown that successful induction of TLSs can take at least one week, and even then, the formed TLSs may not be ideal, with low success rates. This means that TLSs remain extremely challenging to apply in clinical settings. Recent studies have shown promise in utilizing biomaterials in disease, suggesting that leveraging biomaterial versatility to create an environment conducive to TLS formation deserves further investigation. This also forces us to consider ethical issues during the research process. Although our aim is to improve the prognosis of patients by modulating TLSs as a therapeutic tool, their formation is uncontrollable and may cause unpredictable damage to patients during the treatment. Therefore, patients should be fully informed.

## Conclusion

Over the past two decades, the understanding of TLSs has significantly increased. Similar to SLOs, the formation of TLSs is driven by the interaction between LTi cells and LTo cells, with multiple cytokines involved. Among them, mesenchymal cells obtaining LTo features are a prerequisite for triggering TLS formation. Subsequently, chemokines, as the most critical factors for immune cell aggregation, promote lymphocyte recruitment and segregation of B/T cell compartments in TLSs. TLSs are classically defined as lymphoid aggregates formed in non-hematopoietic organs, allowing the generation of effector T cells and effector B cells. We hypothesize that TLSs initiate a faster immune response in the lymphocyte niche. They avoid DCs transporting antigens over long distances and increase contact with T cells to improve antigen presentation efficiency. Meanwhile, TLSs facilitate the penetration of immune cells and cytokines. Consistent with this, “newborn” T cells and B cells in TLSs are present in the local tissue, reducing dependence on naive lymphocytes transported distantly through HEVs. In recent years, the link between TLSs and immunotherapy has gained great attention, and many researchers have realized the broad application of TLSs in immunotherapy, especially anti-tumor immunotherapy. A series of clinical studies have demonstrated that TLSs can significantly enhance the survival of tumor patients, especially as markers of immunotherapy. And many immunotherapies can also promote the formation of TLSs. Therefore, targeting and modulating TLSs will help establish a new immunotherapeutic pathway. However, TLSs are still poorly understood, and their composition, formation, and regulatory pathways need further exploration. In addition, it is still poorly understood whether direct modulation of TLS formation or function by biomaterials or drugs contributes to the clinical prognosis of patients, necessitating enhanced clinical studies.
